# Understanding integrated service delivery: a scoping review of models for noncommunicable disease and mental health interventions in low-and-middle income countries

**DOI:** 10.1186/s12913-023-09072-9

**Published:** 2023-01-30

**Authors:** Alma J. Adler, Laura Drown, Chantelle Boudreaux, Matthew M. Coates, Andrew Marx, Oyetayo Akala, Temo Waqanivalu, Hongyi Xu, Gene Bukhman

**Affiliations:** 1 Division of Global Health Equity, Department of Medicine, Brigham and Women’s Hospital, 75 Francis St, Boston, MA USA; 2 Program in Global Noncommunicable Disease and Social Change, Department of Global Health and Social Medicine, Harvard Medical School, 641 Huntington Ave, Boston, MA USA; 3 Noncommunicable Diseases Department, World Health Organization, 20, Avenue Appia-1211, Geneva, Switzerland

**Keywords:** Noncommunicable diseases, Mental health, Low- and middle-income countries, Integration science, Service delivery, Systematic review

## Abstract

**Background:**

Noncommunicable diseases (NCDs) and mental health conditions represent a growing proportion of disease burden in low- and middle-income countries (LMICs). While past efforts have identified interventions to be delivered across health system levels to address this burden, the challenge remains of how to deliver heterogenous interventions in resource-constrained settings. One possible solution is the Integration of interventions within existing care delivery models. This study reviews and summarizes published literature on models of integrated NCD and mental health care in LMICs.

**Methods:**

We searched Pubmed, African Index Medicus and reference lists to conduct a scoping review of studies describing an integrated model of NCD or neuropsychiatric conditions (NPs) implemented in a LMIC. Conditions of interest were grouped into common and severe NCDs and NPs. We identified domains of interest and types of service integration, conducting a narrative synthesis of study types. Studies were screened and characteristics were extracted for all relevant studies. Results are reported using PRISMA-ScR.

**Results:**

Our search yielded 5004 studies, we included 219 models of integration from 188 studies. Most studies were conducted in middle-income countries, with the majority in sub-Saharan Africa. Health services were offered across all health system levels, with most models implemented at health centers. Common NCDs (including type 2 diabetes and hypertension) were most frequently addressed by these models, followed by common NPs (including depression and anxiety). Conditions and/or services were often integrated into existing primary healthcare, HIV, maternal and child health programs. Services provided for conditions of interest varied and frequency of these services differed across health system levels. Many models demonstrated decentralization of services to lower health system levels, and task shifting to lower cadre providers.

**Conclusions:**

While integrated service design is a promising method to achieve ambitious global goals, little is known about what works, when, and why. This review characterizing care integration programs is an initial step toward developing a structured study of care integration.

**Supplementary Information:**

The online version contains supplementary material available at 10.1186/s12913-023-09072-9.

## Background

Noncommunicable diseases (NCDs) and mental health (MH) conditions account for a large and growing proportion of the disease burden in all regions, including in low- and middle-income countries (LMICs) [1]. The World Health Organization (WHO) identified a core set of evidence-based “best buy” interventions to address these conditions [2, 3]. The best buy interventions are mainly population-level efforts to reduce exposure to certain modifiable risk factors. The best buys also include interventions that must be delivered through the health sector: specifically, multidrug therapy for adults at high risk of cardiovascular events, vaccination for human papillomavirus and hepatitis B virus, as well as cervical cancer screening. The Disease Control Priorities, 3^rd^ edition (DCP3) and the *Lancet* Commission on Reframing NCDs and Injuries for the Poorest Billion (NCDI Poverty Commission) have also identified a much larger set of cost-effective and equitable interventions to address the NCDI burden [4, 5]. In addition to 74 intersectoral policies, the NCDI Poverty Commission identified 183 important health sector interventions including education, medical and surgical treatments, rehabilitation, and palliative care. These interventions require delivery at all health system levels, including the community, primary health centers, secondary-level facilities, and tertiary-level referral centers. Recent estimates by WHO of resource requirements for Universal Health Coverage (UHC) have included many/most of the same interventions highlighted by DCP3 and the NCDI Poverty Commission [6].

The challenge then is how to deliver this heterogenous set of interventions, particularly in health systems with significant human resource and infrastructure constraints. To address this challenge, the NCDI Poverty Commission co-chairs called for a “Science of Integration” to guide the design of integrated care delivery models [7]. The Commission introduced a set of illustrative “integrated care teams” that could deliver sets of interventions requiring similar competencies and infrastructure. The integrated care team concept builds on prior work by WHO to group sets of interventions through the WHO Package of Essential Noncommunicable (PEN) Interventions for Primary Health Care strategy, as well as the Integrated Management of Childhood Illness (IMCI) and Integrated Management of Adult and Adolescent Illness (IMAI) approaches at both primary and secondary health system levels [8–10].

WHO considers integration to be an important strategy for scaling up both NCD and MH treatments and is currently engaged in initiatives to support countries in implementing integrated service delivery and specifically suggests the implementation of mental health services into primary care [11]. In 2016, WHO member states adopted the Framework on integrated people-centered health services, which proposed a set of interdependent strategies to reorient health services towards more integrated and people-centered models of care [12]. Additional WHO efforts include the creation of a UHC compendium and comprehensive guidance on NCD services integration. As part of this initiative on guidance on NCD integrated service delivery, WHO recently commissioned a rapid systematic review which identified areas to consider when planning, implementing, and evaluating NCD integration at primary care level. This was elaborated as the following overarching themes: policy and governance; leadership and transformation management; providers engagement and team care; providers knowledge, skills, and training; intervention compatibility and health system readiness supporting services such as supply system, infrastructure, financial system, human resource management; and health information and monitoring [13].

There is considerable literature aimed at defining and systematizing research on integration, reflecting the range of priorities and objectives embedded in integration efforts [14, 15]. These include, for example, quality improvement initiatives, efforts to create a patient-centered care experience, and efforts to rapidly expand access to services in low-resource settings. Much of this variability is due to the diversity of health system stages, needs, changes and priorities.

While published research exists on service packages, sets of interventions, and models of care, no one, to our knowledge, has systematically reviewed the literature to comprehensively identify different models of service integration. Published literature includes reviews focused on models of integrated NCD care in LMICs [16–24]. Condition of focus for these reviews include type 2 diabetes, hypertension, cervical cancer, and chronic kidney disease [16–20]. Together these studies cover a broad range of conditions, settings, and models of care, but they are each fairly limited in scope. Therefore, a more comprehensive review to examine all integrated models of NCD and MH care implemented in LMICs is needed. In this study, we characterize the published literature regarding delivery models of integrated care to address priority NCDs and MH conditions in LMICs. This review and summary study will enable future work to develop a classification system of dimensions that explain and differentiate models of integrated care. By categorizing delivery models based on this classification system, we will be able to identify patterns and gaps in the focus of NCD and MH research. We will also be able to develop a foundation for comparative analysis regarding the impact of alternative delivery models on the cost and benefits of interventions. Here we answer the questions, what models of integration exist in the literature, and what patterns can we identify in the design and implementation of these models.

## Methods

### Criteria for considering studies for this review

We conducted a scoping review to describe existing models of integrated care. We included all studies published since 2000 describing an integrated model of NCD or MH care implemented in a LMIC. We chose to only include LMICs as we sought to understand the group furthest behind in achievement of UHC. As models of care change over time, we made the decision to only include studies published post 2000. Studies included those describing a new model, as well as those discussing previously implemented models. All study designs – including but not limited to, trials, qualitative analyses, cohort studies, and cross-sectional studies – were included. In randomized control trials testing an integrated model of care versus standard care, we extracted data on the integrated model. Models only focusing on special populations (for example, models only including refugees and internally displaced populations) were excluded as generally these were not representative of the health system. Any study that reported on multiple health system levels where we were unable to differentiate what conditions or services were provided at each level was excluded.

### Search methods for identification of studies

We searched the medicine-focused databases, PubMed on January 14, 2021 and African Index Medicus on March 11, 2021. Search phrases included terms around a) integrated care and patient care teams, b) LMICs, and c) key NCD and MH conditions. The complete search strategy is found in Additional file 1: Appendix A*.* To supplement our search, we also hand-searched reference lists of identified studies and reviews. No limits were applied to the search strategy.

### Data collection and analysis

#### Selection of studies

Data were downloaded and screened in Endnote [25]. Studies were first screened by an initial review of titles and abstracts by two authors (AJA, LD). Twenty percent of studies were double screened. Where applicable, full text was obtained for relevant studies identified through a second screen of studies. During screening we limited our selection to studies published post 2000.

#### Data extraction and management

Data were extracted into a piloted Excel spreadsheet. As more studies were included, new fields were added to the spreadsheets in an iterative process, and we reexamined the original studies to extract additional fields. Characteristics from all studies were initially extracted by one author (LD) and then checked by a second author (CB). Discrepancies were resolved through conversation among three authors (AJA, CB, LD).

In addition to the core functions of integrated care, we also extracted information on domains likely to affect health sector priorities. These included country, income level, setting, if the study was a research study, HIV prevalence, health system level, integration type, primary care provider, services delivered, conditions treated, and patient fees (Table 1) Additional details on key domains are provided in Supplementary Table 1. For studies conducted at the community level, we also extracted effort of provider (full or part time), whether the delivery was mobile, and health care workers compensation (Table 1).

**Table 1:** Study delivery model dimensions extracted with identified typologies

Dimension	Types identified
Health system level	Community
	Health center
	Secondary level facility
	Outpatient
	Inpatient
	Tertiary level facility
	Outpatient
	Inpatient
	Specialty outpatient clinic
Urban or rural	Urban
	Rural
	Peri-urban
	Mixed
Scale	Single center
	Small to medium scale
	Large scale
	National
	Multi-country
Institution	Private
	Public
	Public (with external funding)
	Nongovernmental organization (NGO)
	Faith-based organization (FBO)
Integration type	New care delivery teams
	Description of existing delivery models
	Task redistribution within existing delivery models
	New conditions integrated into existing delivery models
	New services integrated into existing delivery models
	New conditions and services integrated into existing delivery models
Primary provider	Lay staff
	HIV counsellor
	Social worker
	Traditional healer
	Community health worker (CHW)
	Midlevel provider
	Generalist physician
	Specialist physician
	No primary provider
	Multidisciplinary team
	Multi-cadre team
Patients’ fee	Free
	Out of pocket
	Copay
Decentralization	No
	Yes
Task shifting	No
	Yes
Linkage	Counter-referral
	Referral
Mobile	No
	Yes (including community campaigns and mobile clinics)
Effort of primary provider	Part-time
	Full-time
Compensation of primary provider	Salaried
	Fee-for-service
	Volunteer
Service^a^	Health promotion
	Health education
	Screening
	Referral
	Initial diagnosis
	Adherence support
	Peer group facilitation
	Acute care
	Home based care
	Home based visits
	Psychotherapy
	Medication dispensing
	Patient follow-up
	Monitoring
	Medication management
Condition category^a^	Common chronic noncommunicable diseases (NCDs)
	Severe chronic NCDs
	Common neuropsychiatric (NP)
	Severe NP
	Chronic infection
	Acute infection
	Maternal and child health
	Conditions addressed through “primary health care”
	Sense organ

^a^For definitions see supplementary Table 1

Data were extracted to reflect what was written in the published studies, with the exception of provider effort as explained below.

#### Analytic framework

We use the WHO definition of integrated health services to guide our analytic framework: services that are managed and delivered so that people receive a continuum of health promotion, disease prevention, diagnosis, treatment, disease management, rehabilitation, and palliative care services, coordinated across the different levels and sites of care within and beyond the health sector, and according to their needs throughout the life-course [26]. Integration must be reflected in the processes of care delivery, organization of providers and management of services [27]. We prioritized these typologies as they are specifically relevant for expansion of service care delivery. To aid in identifying patterns in model characteristics, countries were classified by geographic region and income group using 2021 World Bank categories. To reflect the particular importance of historic HIV funding on health systems development, we also classified countries according to HIV prevalence, divided into three categories based on UNAIDS classifications (less than one percent prevalence, between one and five percent prevalence, and five or higher percent prevalence) [28].Throughout our results we pool NCDs and MH.

Domains showing our analytic framework are outlined in Table 1.

#### Definitions

Health system level was divided into community, primary health care (generally health centers), secondary care (often referred to as district-level hospitals), and tertiary care (often referred to as provincial and/or central level hospitals). Definitions for services delivered and health condition categories are found in Supplementary Table 1. We renamed MH as neuropsychiatric (NP) to be inclusive of common and severe mental health disorders as well as neurological conditions particularly epilepsy. Provider types included community health workers (CHWs), counsellors, pharmacists, mid-level providers (including nurses and clinical officers), generalist and specialist physicians, multi-cadre teams (including health workers from multiple levels, for example a nurse and a physician), and multidisciplinary teams (including the same cadre health worker from many different disciplines). Institution referred to the nature of the institution offering services.

For each of the studies, we documented what conditions and services are offered through the delivery model. We originally intended to include information about what specific conditions were treated and what services were included prior to the integration. We were unable to extract this data in a consistent way, however, and as a result, we did not include it in the results. Instead, we present data on whether prior services were offered through routine care or vertical programs, such as chronic infectious disease clinics including HIV or MCH clinics.

Three domains that we extracted were only relevant at the community level – mobility, compensation, and effort level. Models were defined as mobile if they were brought into the community regularly or occasionally. Examples of mobile models included community campaigns and mobile clinics. Compensation referred to whether the healthcare workers (generally CHWs) were paid. Categories included volunteer, salaried (either through money or goods), or fee-for-service. We acknowledge that in some cases definitions may differ by country and some CHWs considered to be volunteers in some countries may be paid with goods or other means. Our categorization was restricted to how compensation was reported in the studies. For community level studies, effort level (full or part time) of the primary provider was defined by the healthcare workers (generally CHWs) working either full-time or part-time. This was not generally specified in the papers (70%), but we extrapolated this based on workload and estimation of time spent as specified in the article. We considered any effort that was 20 h per week or more to be full-time.

We additionally examined whether models demonstrated decentralization of health services, defined here as models where new services previously mapped to one level were introduced at a lower health system level.

#### Assessment of risk of bias

Since this study describes what was written describing models of care, it was not feasible to conduct a risk of bias assessment. However, we acknowledge biases are inherent in the overall process and these are described below.

#### Data synthesis

We conducted a narrative synthesis of the types of studies describing integration. We provide percentages of studies that fall into each category.

## Results

### Results of the search

**Fig.1:**
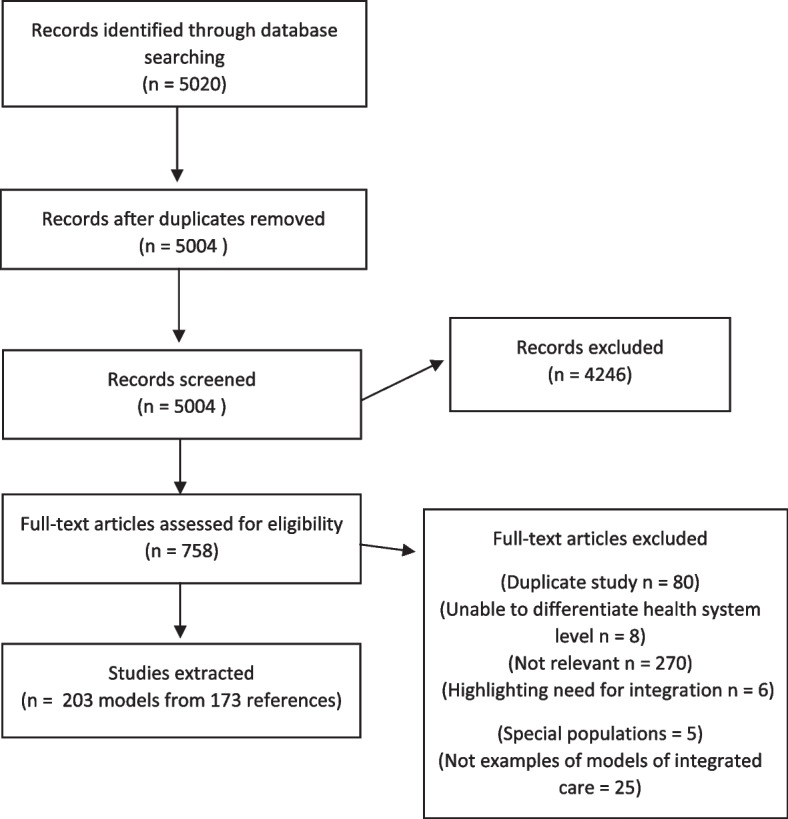
Prisma diagram of included studies

Our initial search yielded 5020 studies, which was reduced to 5004 after deduplication. After title and abstract screening, 758 studies remained. From the studies that were double screened, no papers considered relevant were found to be missing. 271 studies were found to be irrelevant. Reasons for exclusions are found in Fig. 1. Through hand searching and citation searching we identified 18 models from 16 studies. After extraction 219 models from 188 studies remained (Fig. 1, see Additional file 2: Appendix B-F for included studies).

### Settings

Of the 188 overall studies, 29 (15%) were conducted in low-income countries (LICs), 67 (36%) were conducted in lower-middle-income countries (L-MICs), 88 (47%) were conducted in upper-middle-income countries (UMICs), and four (2%) were conducted in multiple countries including a mix of income levels. Ninety (48%) were conducted in sub-Saharan Africa, 34 (18%) in East Asia and the Pacific, 27 (14%) in South Asia, eight (4%) in Europe and Central Asia, four (2%) in the Middle East and North Africa, and 25 (13%) in Latin America and the Caribbean.

Models were categorized by which level of the health system offered services. Of the 219 models, thirty-five were mixed across multiple levels. Based on available information in the studies or other sources, we were able to separate 26 of these studies by the level of health system into multiple levels. Thus, one study that reported on a model in the community, health center and secondary facility, would be included in three models. This led to an additional 31 models being included, for a total of 219 across 188 studies. These included community (*n* = 55, 25%), health center (primary health care) (*n* = 93, 42%), secondary health facilities (*n* = 31, 14%), and tertiary hospitals (*n* = 30, 14%). Ten studies (5%) were in standalone specialty outpatient clinics, which were considered separately.

Among studies that specified where services were delivered, 46 (24%) were conducted in rural settings, 70 (37%) in urban settings, four (2%) in peri-urban or suburban areas, and 31 (16%) in mixed regions. Most studies were conducted at single centers (31%) or on the small to medium scale (47%). The most frequently identified institutions were public (112 studies, 60%) and public with outside support (*n* = 40 studies, 21%). Other models included private (three studies, 2%) nongovernmental organizations (eight studies, 4%), and faith-based organization (two studies, 1%) (Table 2). Nearly all studies found by this search outlined outpatient services.

**Table 2:** Number and percentage of included studies by income level, region, delivery area, scale, institution, research role in delivery model, and condition category

Dimension and category	Number of studies (*N* = 188) n (%)
**Income group**	**n (%)**
Low-income country (LIC)	29 (16)
Lower middle-income country (L-MIC)	67 (34)
Upper middle-income country (UMIC)	88 (48)
Combined	4 (2)
**Region**	**n (%)**
Sub-Saharan Africa	90 (47)
North Africa & Middle East	4 (2)
Europe & Central Asia	8 (5)
South Asia	27 (16)
East Asia & the Pacific	34 (16)
Latin America & Caribbean	25 (14)
**Delivery area**	**n (%)**
Rural	46 (25)
Urban	70 (35)
Peri-urban	4 (2)
Mixed	31 (18)
Not specified	37 (19)
**Scale**	**N %**
Single center	58(30)
Small to medium scale	88 (46)
Large scale	31 (18)
National	8 (5)
Multi-country	3(2)
**Institution**	**n (%)**
Public	112 (62)
Public with outside support	40(21)
Private	3 (2)
Non-governmental organizations	8(3)
Faith based organization	2 (2)
Not specified	23 (11)
**Was the delivery model part of routine care or a research study?**	**n (%)**
Theoretical research protocols (not yet implemented)	17 (10)
Pilot and feasibility studies	57 (27)
Experimental studies	30 (17)
Imbedded or evaluation	84 (47)
**Condition category**	**n (%)**
Common chronic NCDs	118 (64)
Severe chronic NCDs	42 (21)
Common neuropsychiatric	53 (29)
Severe neuropsychiatric	15 (9)
Chronic infection	49 (24)
Acute infection	11 (6)
Maternal and child health	23 (13)
Conditions addressed through “primary health care” not otherwise specified	27 (15)
Sense organ	1 (1)

The majority of studies reported being experimental in nature, including untested protocols (*n* = 17, 9%), pilot studies and feasibility studies (*n* = 57, 30%), or experimental studies (*n* = 30, 16%). Eighty-four were considered to be embedded into the health system, or evaluations of current programs (45%) (Table 2).

Over time, publication of integrated models increased. Our study identified only 16 studies from the ten-year periods between 2000 to 2009 (representing less than three per 1,000,000 studies indexed by Medline during that time), 49 studies from 2010 to 2015 (representing almost 11 per 1,000,000 studies indexed by Medline during that time), 121 studies from 2016 to 2020 (representing almost 27 per 1,000,000 studies indexed by Medline during that time) and two in 2021.

### Integration type

Among the studies reviewed here, 12% described existing models. The remainder documented changes to existing models including: new care teams providing integrated care (26%), task distribution within existing models (5%), new services integrated into existing models (16%), new conditions integrated into existing models (26%) and new services and conditions integrated into existing models (15%). In the community (31%) and tertiary level (30%), new care delivery teams were the most frequently identified model. At health centers (28%) and at the secondary level (35%) new conditions were typically integrated into existing models (Table 3).

**Table 3:**
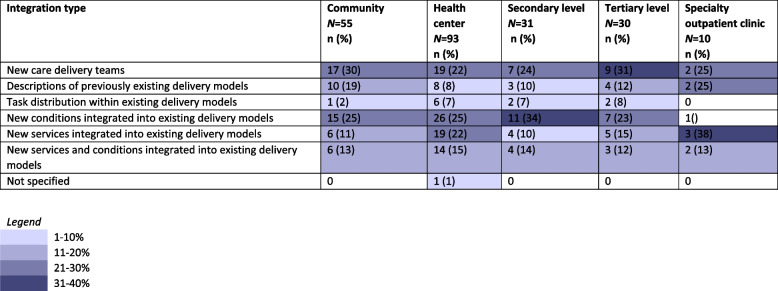
Number and percentage of integration types utilized in each model, stratified by health system level

### Providers

Many types of primary providers were identified as shown in Table 1. We identified two types of studies that did not have a single primary provider: multi-cadre (different cadres within the same general discipline) and multidisciplinary teams (people from multiple disciplines, for example a mental health counselor and an NCD provider). At the community level, CHWs were the most frequent primary provider (47%). Mid-level providers were the most frequently reported primary provider in health centers (42%), secondary facilities (48%) and tertiary facilities (30%) (Table 4). Models utilizing multidisciplinary teams instead of a single primary provider were more likely to focus on a single condition such as diabetes or palliative care (Additional file 2: Appendix B-F).

**Table 4:**
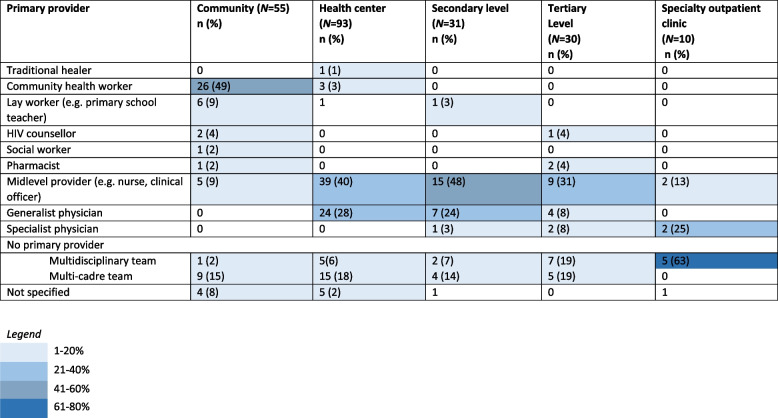
Number and percentage of primary provider categories reported in study models stratified by health system level

### Decentralization and task shifting

Decentralization was identified often in this review, and was particularly at the community level, where services previously found at health care facilities were decentralized to the community (33%), and health centers (18%), where care was decentralized from hospitals. We also found decentralization in 23% of models at secondary facilities. Similarly, 22% of studies reported task shifting from care teams to lower cadre providers in the community, 37% at health centers, and 39% at secondary facilities (Supplementary Table 2).

### Service types

We identified 15 unique service types across the studies. A complete list is found in Supplementary Table 1.

At all health system levels, health education was found in the majority of models. At the community level, most models reported linkage to higher levels of the health system (69%), health education (65%), and screening (62%). Health promotion (40%), adherence support (38%), and home visits (33%) were also frequent. At the health center level, in addition to the services found at the community level, initial diagnosis (48%), patient follow-up (54%), medication dispensing (59%) and some medication management (12%) became more prevalent. There was less health promotion (6%), screening (45%), and home visits (11%). At secondary facilities, initial diagnosis (58%), medication dispensing (77%), patient follow-up (62%), and monitoring (52%) were all found in most studies, with a large proportion reporting medication management (42%) (Table 5).

**Table 5:**
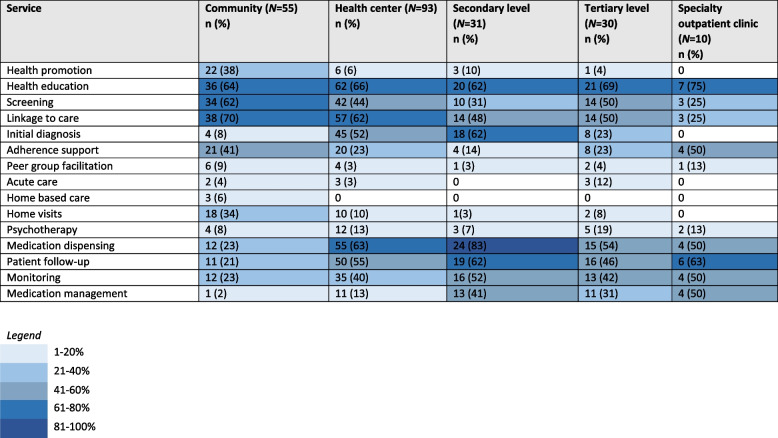
Number and percentage of service type categories reported in study models stratified by health system level

### Conditions treated

Conditions named in the studies are summarized into the following condition types: common chronic NCDs, severe chronic NCDs, common NPs, severe NPs, maternal and child health (MCH), conditions reported in studies as being covered by “primary health care” (PHC), acute infectious diseases, chronic infectious diseases, and sense organ disorders. The specific conditions included within each of these condition types are described in Supplementary Table 1. Common NCDs were the most popular, reported in 118 studies (63%), followed by common NP conditions reported in 53 (28%) studies (Table 2). Conditions included in models stratified by health system level are shown in Table 6.

**Table 6:**
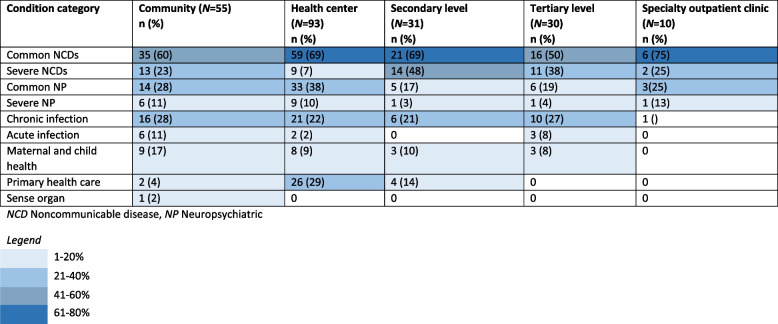
Number and percentage of condition categories included in study models stratified by health system level

The condition categories that most often were found to be integrated together were common NCDs integrated into chronic infection programs. In countries with a low HIV prevalence, however, there were only two studies showing models of integrated services for NCDs or NP and chronic infections. In studies conducted in these countries, models frequently showed services for NCDs or NPs being integrated into MCH or PHC services (23 studies, 25%). In mid-range HIV prevalence countries, NCDs and NPs (common and severe) were often integrated into MCH or PHC (16 studies, 53%), but also into chronic infections (12 models, 40%). In high HIV prevalence countries, 35 studies (67%) included NCDs and NP being integrated into chronic infections programs, but only 14 (27%) into MCH and PHC (Supplementary Table 3).

Studies in LICs tended to have a higher percentage of models including more severe conditions. Forty-one percent of studies conducted in LICs included severe NCDs, compared to 25% in L-MICs, and 13% in UMICs. Similarly, 10% of studies in LICs included severe NPs, compared to 7% in L-MICs and 7% in UMICs. The percentage of studies that included common NCDs and NPs was roughly equal across income levels (Supplementary Table 4).

### Other domains

Only one-third of studies (33%) documented the cost of care. A majority of the studies took place in the public/government-run health system and are subject to national policies. Among the studies reporting cost of care, the majority specified that at least some services or medications are offered free of charge, while some reported out-of-pocket payments, including fee-for-service, co-pays, or consultation fees (Supplementary Table 5).

For domains unique to community-based studies, ten studies (18%) were in mobile clinics. The majority of studies (69%) did not report on compensation, but of those that did, nine (16%) reported CHWs as volunteers, and the rest reported compensation as a salary (11%) or as fee for service (4%). In nine (16%) studies, CHWs worked part time, and in seven (13%) they worked full time (Supplementary Table 6).

## Discussion

### Summary of evidence

We identified 219 unique service delivery models from 188 studies in 44 countries in this review. The largest number of studies were conducted in UMICs and involved services delivered at primary health centers. At all levels of the health system except for community, mid-level providers were the most frequent primary provider. Many delivery models included the decentralization of services initially only available at higher levels of the health system into lower-level facilities, often utilizing mid-level providers. Task shifting and task redistribution were also typical approaches. In high-HIV-prevalence countries, NCD and NP programs were often integrated into HIV programs; in low-HIV-prevalence countries, NCD and NP programs were often integrated into MCH/PHC programs; and in medium-HIV-prevalence countries, NCD and NP programs were integrated into both HIV and MCH/PHC programs.

Interestingly, severe (as compared to common) NCDs and NPs were more likely to be included in studies conducted in LICs. This might be due to better-resourced countries already having these services in place, without the need for integrated models, or existing integrated models not being reported in the literature. Publication bias also probably accounts for some of this finding—programs targeting severe diseases in LICs may be considered more innovative and publishable than in HICs.

The goals and the structure of new service delivery design will, in many ways, reflect the realities of a given health system. In areas with significant funding dedicated to HIV prevention and care, studies were likely to highlight opportunities to leverage these systems, which may reflect complementarities in skills, infrastructure and systems support needed to provide continuity in chronic care. Thus, researchers from Tanzania, South Africa and Malawi all described efforts to integrate NCD care into existing chronic care clinics originally launched to provide HIV services [29–31].

Integration design must also reflect the local realities of a given clinic. Most broadly, efforts to introduce new services into existing specialized programs are likely to differ from those targeting general primary care. Operations at specialized clinics (for example HIV, MCH, Immunization/IMCI) are often adapted to role differentiation and task distribution, particularly in multi-cadre teams. New services delivered in such clinics are often protocolized services that can be broken into pieces and distributed across staff. In these cases, integration was often framed as an effort to leverage access to the particular population treated within the specialized clinic. For example, multiple researchers sought to integrate MH/depression screening into adolescent and maternal health services [32–34].

### What this study adds

While a number of other researchers have conducted reviews around models of integrated care, most have conducted narrower reviews focusing on only one condition such as type 2 diabetes, chronic kidney disease, cervical cancer, or hypertension [16–20]. Others were only interested in NCDs integrated into HIV care or MCH [21–23]. A review of NCD and HIV integration in low-income countries identified three models of integration—NCD services integrated into facilities already providing HIV care, HIV care integrated into PHC where NCD services were already offered, and simultaneous introduced of HIV and NCD services [22]. Like our review, this study reported a large variety of NCD services integrated with HIV in these models [22]. Unlike our study, this review did not differentiate between added services and conditions. We found a number of reviews looking at integrated MH [21, 24]. Some studies were restricted to L-MICs or LICs or one specific country [20–24]. To our knowledge, this is the first study to look at all integrated models of NCDs and NPs in all non-HIC countries, and is the most comprehensive review done to this time.

### Limitations of the review

A major limitation of any review, particularly of this type, is that it is limited to what is found in the published literature, which may over-represent more successful or better-funded efforts, particularly in better-resourced settings. We are sure that there are many examples of integration that were not included in this study as they were not published, or found by the search. Search results were likely limited by the small number of regional databases utilized. Our study reports a high percentage of pilot or experimental studies, with fewer studies with long-term follow-up, likely due to experimental and pilot studies being seen as more “publishable” than studies reporting monitoring of services. The institutions identified in this review were not necessarily representative of the picture in LMICs. We identified three studies that were completely in private institutions, whereas in reality many LMICs have mixed health systems. Because our findings were based on the published literature, we were restricted to services and conditions explicitly reported in the publications, meaning that we may have missed or underrepresented conditions and services that were offered in the programs, but not mentioned in these studies. The majority of studies were lacking information on compensation, and fees, so what we present here may not be an accurate representation of what is actually happening on the sites. Without a good description of what services were offered and conditions were treated prior to integration, it is challenging to describe the overall model. Terminology can vary among countries, and while the authors made every attempt to clarify for the classification, complete data was often lacking. Interventions are often complex, and in many cases had to be simplified to fit the rubric. The notable lack of studies reporting services around acute care and surgery points to a gap in the search strategy, so subsequent studies will need to address these services.

Finally, the authors here were also involved in some of the included studies. As a result, those particular studies may have more complete reporting, as we have more intimate knowledge of these delivery models, however we made every effort to not include information not explicitly stated in the paper.

### Implications for future research and implementation

This study is the first step in creating a classification system for integrated care delivery. Future research will take these studies and create models of integrated care and a full classification system. This study does not attempt to examine the effectiveness of the different models of care. Once the classification system is complete, however, future research will analyze the effectiveness of different models and the evolution of the model over time. Other future research can be directed towards follow-up studies on pilots reported in this study and research the characteristics of sustainable ones. Finally, we identified several studies of broader scope where we were unable to differentiate between specific services provided at different levels of the health care system, and future studies can analyze more of the “district” level models implementing integration across multiple levels and population impact.

Moving forward, integrated NCD program design efforts must reflect health system needs and can build on historic health system priorities or leverage typical targets of funds including hypertension or other popular NCDs as platforms for adding in more complex conditions and services.

Finally, we appeal to the academic community to consider the fields and domains discussed in this study both when designing integrated studies as well as when they are reporting on them. Items such as user fees, provider compensation, training, and prerequisite qualifications are paramount to the success of any program, and the paucity of data in these fields will impair researchers and implementers from learning from their experiences. We will also be able to develop a foundation for comparative analysis regarding the impact of alternative delivery models on the cost and benefits of interventions.

## Conclusion

Gaps in NCD care/UHC require the implementation of a heterogenous group of conditions and interventions. Achieving global goals will require the efficient use of limited resources. While integrated service design is seen as a promising method to achieve this goal, evidence in the existing published literature about what works, when, and why is sparse. This study is the beginning of a program aimed at developing a structured study of care integration. Here, we have characterized integrated programs across a range of settings. Future work will include developing a classification system to define, understand, and differentiate models of integration. By categorizing delivery models based on this classification system, we will be able to identify patterns and gaps in the focus of NCD implementation research on service delivery. We will also be able to develop a foundation for comparative analysis regarding the impact of alternative delivery models on the cost and benefits of interventions.

## Supplementary Information

**Additional file 1: Appendix A.**

**Additional file 2:** **Appendix B.** Delivery models identified at the community level. **Appendix C.** Delivery models identified at health centers. **Appendix D**. Delivery models identified at secondary level facilities. **Appendix E.** Delivery models identified at tertiary level institutions. **Appendix F.** Studies identified at specialty outpatient clinics.

**Additional file 3:** **Supplementary Table 1.** Definitions of identified services and disease condition categories (consistent with WHO Integrated health services definition). **Supplementary Table 2.** Number and percentage of models that include decentralization and/or task shifting stratified by health system level. **Supplementary Table 3.** Number and percentage of noncommunicable disease or neuropsychiatric models integrated into chronic infection or MCH/PHC models stratified by HIV prevalence in the country. **Supplementary Table 4.** Number and percentage of common and severe condition categories reported in study models within each country income group. **Supplementary Table 5.** Number and percentage of studies reporting how care is paid for reported in each study model, stratified by health system level. **Supplementary Table 6.** Number and percentage of additional domains identified in community-based studies (*N*=55) including if model was mobile, compensation of primary provider, and primary provider effort.

## Data Availability

All data included here are from publicly available sources. Our search strategy is included in Additional file 1: Appendix A.

## Abbreviations

CHW: Community health worker
DCP3: Disease Control Priorities, 3rd edition
HICs: High-income countries
HIV: Human immunodeficiency virus
IMAI: Integrated Management of Adult and Adolescent Illness
IMCI: Integrated Management of Childhood Illness
LICs: Low-income countries
LMICs: Low- and middle-income countries
L-MIC: Lower-middle income countries
MCH: Maternal and child health
MH: Mental health
NCD: Noncommunicable diseases
NP: Neuropsychiatric
PEN: Package of Essential Noncommunicable
PHC: Primary health care
UHC: Universal Health Coverage
UMICs: Upper middle-income countries
WHO: World Health Organization
