# Supplementary material for: Understanding integrated service delivery: a scoping review of models for noncommunicable disease and mental health interventions in low-and-middle income countries
Source: BMC Health Serv Res. 2023 Jan 30;23:99. doi: 10.1186/s12913-023-09072-9 (PMC9885613; doi:10.1186/s12913-023-09072-9)
Supplement: Supplementary file 1 — Additional file 1: Appendix A. [file 12913_2023_9072_MOESM1_ESM.docx]

**Appendix A**

**Search Strategy: (colored sections combined with ands)**

**[NCD portion]**

**NCDs**

**((("Noncommunicable"[All Fields] OR "non-communicable"[All Fields] OR ("chronic"[All Fields] OR "chronical"[All Fields] OR "chronically"[All Fields] OR "chronicities"[All Fields] OR "chronicity"[All Fields] OR "chronicization"[All Fields] OR "chronics"[All Fields])) AND ("disease"[MeSH Terms] OR "disease"[All Fields] OR "diseases"[All Fields] OR "diseased"[All Fields] OR ("disease"[MeSH Terms] OR "disease"[All Fields] OR "diseases"[All Fields] OR "disease s"[All Fields] OR "diseased"[All Fields]))) OR "NCD"[All Fields] OR "NCDs"[All Fields] OR "NCDI"[All Fields] OR "NCDIs"[All Fields])**

**OR**

**Diabetes**

**("diabete"[All Fields] OR "diabetes mellitus"[MeSH Terms] OR ("diabetes"[All Fields] AND "mellitus"[All Fields]) OR "diabetes mellitus"[All Fields] OR "diabetes"[All Fields] or "diabetic"[All Fields] OR "diabetics"[All Fields] OR "diabets"[All Fields] OR "T2D"[All Fields] OR "T1D"[All Fields])**

**OR**

**CVD**

**("CVD"[All Fields] OR ("cardiovascular diseases"[MeSH Terms] OR ("cardiovascular"[All Fields] AND "diseases"[All Fields]) OR "cardiovascular diseases"[All Fields] OR ("cardiovascular"[All Fields] AND "disease"[All Fields]) OR "cardiovascular disease"[All Fields]) OR ("hypertense"[All Fields] OR "hypertension"[MeSH Terms] OR "hypertension"[All Fields] OR "hypertensions"[All Fields] OR "hypertensive"[All Fields] OR "hypertensives"[All Fields]) OR "hypertensive heart disease"[All Fields] OR ("heart failure"[MeSH Terms] OR ("heart"[All Fields] AND "failure"[All Fields]) OR "heart failure"[All Fields]) OR ("rheumatic heart disease"[MeSH Terms] OR ("rheumatic"[All Fields] AND "heart"[All Fields] AND "disease"[All Fields]) OR "rheumatic heart disease"[All Fields]) OR "rhd"[All Fields] OR ("pharyngitis"[MeSH Terms] OR "pharyngitis"[All Fields]) OR ("cardiomyopathie"[All Fields] OR "cardiomyopathies"[MeSH Terms] OR "cardiomyopathies"[All Fields] OR "cardiomyopathy"[All Fields]) OR ("cardiomyopathie"[All Fields] OR "cardiomyopathies"[MeSH Terms] OR "cardiomyopathies"[All Fields] OR "cardiomyopathy"[All Fields]))**

**OR**

**Respiratory**

**("asthma"[MeSH Terms] OR "asthma"[All Fields] OR "asthmas"[All Fields] OR ( ("chronic"[All Fields] AND "respiratory"[All Fields] AND "disease"[All Fields]) OR "chronic respiratory disease"[All Fields]) OR ("pulmonary disease, chronic obstructive"[MeSH Terms] OR "chronic obstructive pulmonary disease"[All Fields] OR ("chronic"[All Fields] AND "obstructive"[All Fields] AND "pulmonary"[All Fields] AND "disease"[All Fields])) OR ("pulmonary disease, chronic obstructive"[MeSH Terms] OR ("pulmonary"[All Fields] AND "disease"[All Fields] AND "chronic"[All Fields] AND "obstructive"[All Fields]) OR "chronic obstructive pulmonary disease"[All Fields] OR "copd"[All Fields]))**

**Or**

**Cervical and Breast Cancer**

**("uterine cervical neoplasms"[MeSH Terms] OR ("uterine"[All Fields] AND "cervical"[All Fields] AND "neoplasms"[All Fields]) OR "uterine cervical neoplasms"[All Fields] OR ("cervical"[All Fields] AND "cancer"[All Fields]) OR "cervical cancer"[All Fields] OR ("breast neoplasms"[MeSH Terms] OR ("breast"[All Fields] AND "neoplasms"[All Fields]) OR "breast neoplasms"[All Fields] OR ("breast"[All Fields] AND "cancer"[All Fields]) OR "breast cancer"[All Fields]))**

**OR**

**Palliative care**

**("palliative care"[MeSH Terms] OR ("palliative"[All Fields] AND "care"[All Fields]) OR "palliative care"[All Fields])**

**OR**

**Mental Health**

**("mental health"[MeSH Terms] OR ("mental"[All Fields] AND "health"[All Fields]) OR "mental health"[All Fields] OR ("psychotic disorders"[MeSH Terms] OR ("psychotic"[All Fields] AND "disorders"[All Fields]) OR "psychotic disorders"[All Fields] OR "psychosis"[All Fields]) OR ("bipolar"[All Fields] OR "bipolarity"[All Fields] OR "bipolarization"[All Fields]) OR ("depressed"[All Fields] OR "depression"[MeSH Terms] OR "depression"[All Fields] OR "depressive disorder"[MeSH Terms] OR "depressive disorder"[All Fields] OR "depressivity"[All Fields] OR "depressive"[All Fields]))**

**OR**

**Sickle cell**

**(("sickle"[All Fields] OR "sickled"[All Fields]) AND ("cells"[MeSH Terms] OR "cells"[All Fields] OR "cell"[All Fields])) OR ("anemia, sickle cell"[MeSH Terms] OR ("anemia"[All Fields] AND "sickle"[All Fields] AND "cell"[All Fields]) OR "sickle cell anemia"[All Fields] OR ("sickle"[All Fields] AND "cell"[All Fields] AND "disease"[All Fields]) OR "sickle cell disease"[All Fields])**

**Delivery models**

("delivery of health care, integrated"[MeSH Terms] OR "patient care team"[MeSH Terms] OR "integrated chronic disease management model"[All Fields] OR "Integrated Health Care Systems"[All Fields] OR "Integrated Delivery Systems"[All Fields] OR "Delivery System"[All Fields] OR "Integrated Delivery System"[All Fields] OR "systems integrated delivery"[All Fields] OR "service delivery model"[All Fields] OR "integrated approach"[All Fields] OR "integrated care"[All Fields] or “implementation research”)

OR

(("ncd"[All Fields] or “noncommunicable disease”) AND ("primary health care"[MeSH Terms] OR ("primary"[All Fields] AND "health"[All Fields] AND "care"[All Fields]) OR "primary health care"[All Fields]))

or

("integrability"[All Fields] OR "integrable"[All Fields] OR "integral"[All Fields] OR "integrally"[All Fields] OR "integrals"[All Fields] OR "integrant"[All Fields] OR "integrants"[All Fields] OR "integrate"[All Fields] OR "integrated"[All Fields] OR "integrates"[All Fields] OR "integrating"[All Fields] OR "integration"[All Fields] OR "integrational"[All Fields] OR "integrations"[All Fields] OR "integrative"[All Fields] OR "integratively"[All Fields] OR "integrator"[All Fields] OR "integrators"[All Fields]) AND "NCD"[All Fields]

OR

("ncd"[All Fields] OR ("noncommunicable diseases"[MeSH Terms] OR ("noncommunicable"[All Fields] AND "diseases"[All Fields]) OR "noncommunicable diseases"[All Fields] OR ("noncommunicable"[All Fields] AND "disease"[All Fields]) OR "noncommunicable disease"[All Fields])) AND ("hiv"[MeSH Terms] OR "hiv"[All Fields])

**(Low and Middle income countries)**

"afghanistan"[MeSH Terms] OR "afghanistan"[All Fields] OR "afghanistan s"[All Fields] OR ("angola"[MeSH Terms] OR "angola"[All Fields] OR "angola s"[All Fields]) OR ("albania"[MeSH Terms] OR "albania"[All Fields]) OR ("argentina"[MeSH Terms] OR "argentina"[All Fields] OR "argentina s"[All Fields] OR "argentinae"[All Fields]) OR ("armenia"[MeSH Terms] OR "armenia"[All Fields]) OR ("american samoa"[MeSH Terms] OR ("american"[All Fields] AND "samoa"[All Fields]) OR "american samoa"[All Fields]) OR ("azerbaijan"[MeSH Terms] OR "azerbaijan"[All Fields]) OR ("burundi"[MeSH Terms] OR "burundi"[All Fields]) OR ("benin"[MeSH Terms] OR "benin"[All Fields] OR "benin s"[All Fields]) OR ("burkina faso"[MeSH Terms] OR ("burkina"[All Fields] AND "faso"[All Fields]) OR "burkina faso"[All Fields]) OR ("bangladesh"[MeSH Terms] OR "bangladesh"[All Fields] OR "bangladesh s"[All Fields]) OR ("bulgaria"[MeSH Terms] OR "bulgaria"[All Fields]) OR ("bosnia and herzegovina"[MeSH Terms] OR ("bosnia"[All Fields] AND "herzegovina"[All Fields]) OR "bosnia and herzegovina"[All Fields] OR "bosnia"[All Fields]) OR ("republic of belarus"[MeSH Terms] OR ("republic"[All Fields] AND "belarus"[All Fields]) OR "republic of belarus"[All Fields] OR "belarus"[All Fields]) OR ("belize"[MeSH Terms] OR "belize"[All Fields]) OR ("bolivia"[MeSH Terms] OR "bolivia"[All Fields]) OR ("brazil"[MeSH Terms] OR "brazil"[All Fields] OR "brazil s"[All Fields] OR "brazils"[All Fields]) OR ("bhutan"[MeSH Terms] OR "bhutan"[All Fields] OR "bhutan s"[All Fields]) OR ("botswana"[MeSH Terms] OR "botswana"[All Fields] OR "botswana s"[All Fields]) OR ("central african republic"[MeSH Terms] OR ("central"[All Fields] AND "african"[All Fields] AND "republic"[All Fields]) OR "central african republic"[All Fields]) OR ("china"[MeSH Terms] OR "china"[All Fields] OR "china s"[All Fields] OR "chinas"[All Fields]) OR ("cote d ivoire"[MeSH Terms] OR ("cote"[All Fields] AND "d ivoire"[All Fields]) OR "cote d ivoire"[All Fields]) OR ("cameroon"[MeSH Terms] OR "cameroon"[All Fields] OR "cameroons"[All Fields] OR "cameroon s"[All Fields]) OR ("congo"[MeSH Terms] OR "congo"[All Fields]) OR "Democratic Republic of the Congo"[All Fields] OR ("colombia"[MeSH Terms] OR "colombia"[All Fields] OR "colombia s"[All Fields]) OR ("comoros"[MeSH Terms] OR "comoros"[All Fields] OR "comoro"[All Fields]) OR ("cabo verde"[MeSH Terms] OR ("cabo"[All Fields] AND "verde"[All Fields]) OR "cabo verde"[All Fields]) OR ("costa rica"[MeSH Terms] OR ("costa"[All Fields] AND "rica"[All Fields]) OR "costa rica"[All Fields]) OR ("cuba"[MeSH Terms] OR "cuba"[All Fields]) OR ("djibouti"[MeSH Terms] OR "djibouti"[All Fields]) OR ("dominica"[MeSH Terms] OR "dominica"[All Fields]) OR ("dominican republic"[MeSH Terms] OR ("dominican"[All Fields] AND "republic"[All Fields]) OR "dominican republic"[All Fields]) OR ("algeria"[MeSH Terms] OR "algeria"[All Fields]) OR ("ecuador"[MeSH Terms] OR "ecuador"[All Fields] OR "ecuador s"[All Fields]) OR ("egypt"[MeSH Terms] OR "egypt"[All Fields] OR "egypt s"[All Fields]) OR ("eritrea"[MeSH Terms] OR "eritrea"[All Fields]) OR ("ethiopia"[MeSH Terms] OR "ethiopia"[All Fields] OR "ethiopia s"[All Fields]) OR ("fiji"[MeSH Terms] OR "fiji"[All Fields]) OR ("micronesia"[MeSH Terms] OR "micronesia"[All Fields]) OR ("gabon"[MeSH Terms] OR "gabon"[All Fields]) OR ("georgia"[MeSH Terms] OR "georgia"[All Fields] OR "georgia republic"[MeSH Terms] OR ("georgia"[All Fields] AND "republic"[All Fields]) OR "georgia republic"[All Fields] OR "georgia s"[All Fields]) OR ("ghana"[MeSH Terms] OR "ghana"[All Fields] OR "ghana s"[All Fields]) OR ("guinea"[MeSH Terms] OR "guinea"[All Fields] OR "guinea s"[All Fields] OR "guineas"[All Fields]) OR ("gambia"[MeSH Terms] OR "gambia"[All Fields] OR "gambia s"[All Fields]) OR ("guinea bissau"[MeSH Terms] OR "guinea bissau"[All Fields] OR ("guinea"[All Fields] AND "bissau"[All Fields]) OR "guinea bissau"[All Fields]) OR ("equatorial guinea"[MeSH Terms] OR ("equatorial"[All Fields] AND "guinea"[All Fields]) OR "equatorial guinea"[All Fields]) OR ("grenada"[MeSH Terms] OR "grenada"[All Fields]) OR ("guatemala"[MeSH Terms] OR "guatemala"[All Fields] OR "guatemala s"[All Fields]) OR ("guyana"[MeSH Terms] OR "guyana"[All Fields]) OR ("honduras"[MeSH Terms] OR "honduras"[All Fields]) OR ("haiti"[MeSH Terms] OR "haiti"[All Fields] OR "haiti s"[All Fields]) OR ("indonesia"[MeSH Terms] OR "indonesia"[All Fields] OR "indonesia s"[All Fields] OR "indonesias"[All Fields]) OR ("india"[MeSH Terms] OR "india"[All Fields] OR "india s"[All Fields] OR "indias"[All Fields]) OR ("iran"[MeSH Terms] OR "iran"[All Fields]) OR ("iraq"[MeSH Terms] OR "iraq"[All Fields]) OR ("jamaica"[MeSH Terms] OR "jamaica"[All Fields] OR "jamaica s"[All Fields]) OR ("jordan"[MeSH Terms] OR "jordan"[All Fields]) OR ("kazakhstan"[MeSH Terms] OR "kazakhstan"[All Fields] OR "kazakhstan s"[All Fields]) OR ("kenya"[MeSH Terms] OR "kenya"[All Fields] OR "kenya s"[All Fields]) OR ("kyrgyzstan"[MeSH Terms] OR "kyrgyzstan"[All Fields] OR ("kyrgyz"[All Fields] AND "republic"[All Fields]) OR "kyrgyz republic"[All Fields]) OR ("cambodia"[MeSH Terms] OR "cambodia"[All Fields] OR "cambodia s"[All Fields]) OR ("micronesia"[MeSH Terms] OR "micronesia"[All Fields] OR "kiribati"[All Fields]) OR ("laos"[MeSH Terms] OR "laos"[All Fields] OR ("lao"[All Fields] AND "people s"[All Fields] AND "democratic"[All Fields] AND "republic"[All Fields]) OR "lao people s democratic republic"[All Fields]) OR ("laos"[MeSH Terms] OR "laos"[All Fields]) OR ("lebanon"[MeSH Terms] OR "lebanon"[All Fields] OR "lebanon s"[All Fields]) OR ("liberia"[MeSH Terms] OR "liberia"[All Fields] OR "liberia s"[All Fields]) OR ("libya"[MeSH Terms] OR "libya"[All Fields]) OR ("saint lucia"[MeSH Terms] OR ("saint"[All Fields] AND "lucia"[All Fields]) OR "saint lucia"[All Fields] OR ("st"[All Fields] AND "lucia"[All Fields]) OR "st lucia"[All Fields]) OR ("sri lanka"[MeSH Terms] OR ("sri"[All Fields] AND "lanka"[All Fields]) OR "sri lanka"[All Fields]) OR ("lesotho"[MeSH Terms] OR "lesotho"[All Fields]) OR ("morocco"[MeSH Terms] OR "morocco"[All Fields]) OR ("moldova"[MeSH Terms] OR "moldova"[All Fields]) OR ("madagascar"[MeSH Terms] OR "madagascar"[All Fields] OR "madagascar s"[All Fields]) OR ("indian ocean islands"[MeSH Terms] OR ("indian"[All Fields] AND "ocean"[All Fields] AND "islands"[All Fields]) OR "indian ocean islands"[All Fields] OR "maldives"[All Fields] OR "maldive"[All Fields]) OR ("mexico"[MeSH Terms] OR "mexico"[All Fields] OR "mexico s"[All Fields] OR "mexicos"[All Fields]) OR ("micronesia"[MeSH Terms] OR "micronesia"[All Fields] OR ("marshall"[All Fields] AND "islands"[All Fields]) OR "marshall islands"[All Fields]) OR ("republic of north macedonia"[MeSH Terms] OR ("republic"[All Fields] AND "north"[All Fields] AND "macedonia"[All Fields]) OR "republic of north macedonia"[All Fields] OR ("north"[All Fields] AND "macedonia"[All Fields]) OR "north macedonia"[All Fields]) OR ("mali"[MeSH Terms] OR "mali"[All Fields]) OR ("myanmar"[MeSH Terms] OR "myanmar"[All Fields] OR "myanmar s"[All Fields] OR "myanmars"[All Fields]) OR ("montenegro"[MeSH Terms] OR "montenegro"[All Fields]) OR ("mongolia"[MeSH Terms] OR "mongolia"[All Fields] OR "mongolia s"[All Fields]) OR ("mozambique"[MeSH Terms] OR "mozambique"[All Fields] OR "mozambique s"[All Fields]) OR ("mauritania"[MeSH Terms] OR "mauritania"[All Fields]) OR ("malawi"[MeSH Terms] OR "malawi"[All Fields] OR "malawi s"[All Fields]) OR ("malaysia"[MeSH Terms] OR "malaysia"[All Fields] OR "malaysia s"[All Fields]) OR ("namibia"[MeSH Terms] OR "namibia"[All Fields]) OR ("niger"[MeSH Terms] OR "niger"[All Fields]) OR ("nigeria"[MeSH Terms] OR "nigeria"[All Fields] OR "nigeria s"[All Fields]) OR ("nicaragua"[MeSH Terms] OR "nicaragua"[All Fields] OR "nicaragua s"[All Fields]) OR ("nepal"[MeSH Terms] OR "nepal"[All Fields] OR "nepal s"[All Fields]) OR ("pakistan"[MeSH Terms] OR "pakistan"[All Fields] OR "pakistan s"[All Fields]) OR ("peru"[MeSH Terms] OR "peru"[All Fields]) OR ("philippine"[All Fields] OR "philippines"[MeSH Terms] OR "philippines"[All Fields]) OR ("papua new guinea"[MeSH Terms] OR ("papua"[All Fields] AND "new"[All Fields] AND "guinea"[All Fields]) OR "papua new guinea"[All Fields]) OR ("democratic people s republic of korea"[MeSH Terms] OR ("democratic"[All Fields] AND "people s"[All Fields] AND "republic"[All Fields] AND "korea"[All Fields]) OR "democratic people s republic of korea"[All Fields]) OR ("paraguai"[All Fields] OR "paraguay"[MeSH Terms] OR "paraguay"[All Fields]) OR "Gaza"[All Fields] OR ("russia"[MeSH Terms] OR "russia"[All Fields] OR ("russian"[All Fields] AND "federation"[All Fields]) OR "russian federation"[All Fields]) OR ("russia"[MeSH Terms] OR "russia"[All Fields] OR "russia s"[All Fields] OR "russias"[All Fields]) OR ("rwanda"[MeSH Terms] OR "rwanda"[All Fields] OR "rwanda s"[All Fields]) OR ("sudan"[MeSH Terms] OR "sudan"[All Fields] OR "sudans"[All Fields] OR "sudan s"[All Fields]) OR ("senegal"[MeSH Terms] OR "senegal"[All Fields] OR "senegal s"[All Fields]) OR ("melanesia"[MeSH Terms] OR "melanesia"[All Fields] OR ("solomon"[All Fields] AND "islands"[All Fields]) OR "solomon islands"[All Fields]) OR ("sierra leone"[MeSH Terms] OR ("sierra"[All Fields] AND "leone"[All Fields]) OR "sierra leone"[All Fields]) OR ("el salvador"[MeSH Terms] OR ("el"[All Fields] AND "salvador"[All Fields]) OR "el salvador"[All Fields]) OR ("somalia"[MeSH Terms] OR "somalia"[All Fields]) OR ("serbia"[MeSH Terms] OR "serbia"[All Fields]) OR ("south sudan"[MeSH Terms] OR ("south"[All Fields] AND "sudan"[All Fields]) OR "south sudan"[All Fields]) OR ("Sao"[All Fields] AND "Tome"[All Fields]) OR ("suriname"[MeSH Terms] OR "suriname"[All Fields] OR "surinam"[All Fields]) OR ("eswatini"[MeSH Terms] OR "eswatini"[All Fields]) OR ("syria"[MeSH Terms] OR "syria"[All Fields] OR ("syrian"[All Fields] AND "arab"[All Fields] AND "republic"[All Fields]) OR "syrian arab republic"[All Fields]) OR ("syria"[MeSH Terms] OR "syria"[All Fields] OR "syria s"[All Fields]) OR ("chad"[MeSH Terms] OR "chad"[All Fields]) OR ("togo"[MeSH Terms] OR "togo"[All Fields]) OR ("thailand"[MeSH Terms] OR "thailand"[All Fields] OR "thailand s"[All Fields]) OR ("tajikistan"[MeSH Terms] OR "tajikistan"[All Fields]) OR ("turkmenistan"[MeSH Terms] OR "turkmenistan"[All Fields]) OR ("timor leste"[MeSH Terms] OR "timor leste"[All Fields] OR ("timor"[All Fields] AND "leste"[All Fields]) OR "timor leste"[All Fields]) OR ("tonga"[MeSH Terms] OR "tonga"[All Fields] OR "tonga s"[All Fields]) OR ("tunisia"[MeSH Terms] OR "tunisia"[All Fields]) OR ("turkey"[MeSH Terms] OR "turkey"[All Fields] OR "turkey s"[All Fields] OR "turkeys"[MeSH Terms] OR "turkeys"[All Fields]) OR ("micronesia"[MeSH Terms] OR "micronesia"[All Fields] OR "tuvalu"[All Fields]) OR ("tanzania"[MeSH Terms] OR "tanzania"[All Fields] OR "tanzania s"[All Fields]) OR ("uganda"[MeSH Terms] OR "uganda"[All Fields] OR "uganda s"[All Fields]) OR ("ukraine"[MeSH Terms] OR "ukraine"[All Fields] OR "ukraine s"[All Fields]) OR ("uzbekistan"[MeSH Terms] OR "uzbekistan"[All Fields]) OR (vincent, st[Investigator] OR st vincent[Author] OR st vincent[Investigator]) OR ("saint vincent and the grenadines"[MeSH Terms] OR ("saint"[All Fields] AND "vincent"[All Fields] AND "grenadines"[All Fields]) OR "saint vincent and the grenadines"[All Fields] OR "grenadines"[All Fields]) OR ("venezuela"[MeSH Terms] OR "venezuela"[All Fields] OR "venezuela s"[All Fields]) OR ("vietnam"[MeSH Terms] OR "vietnam"[All Fields] OR "vietnam s"[All Fields]) OR ("vanuatu"[MeSH Terms] OR "vanuatu"[All Fields]) OR ("samoa"[MeSH Terms] OR "samoa"[All Fields] OR "samoas"[All Fields]) OR ("kosovo"[MeSH Terms] OR "kosovo"[All Fields] OR "kosovo s"[All Fields]) OR ("yemen"[MeSH Terms] OR "yemen"[All Fields]) OR ("south africa"[MeSH Terms] OR ("south"[All Fields] AND "africa"[All Fields]) OR "south africa"[All Fields]) OR ("zambia"[MeSH Terms] OR "zambia"[All Fields] OR "zambia s"[All Fields]) OR ("zimbabwe"[MeSH Terms] OR "zimbabwe"[All Fields] OR "zimbabwe s"[All Fields]) OR (("poverty"[MeSH Terms] OR "poverty"[All Fields] OR ("low"[All Fields] AND "income"[All Fields]) OR "low income"[All Fields]) AND ("countries"[All Fields] OR "country"[All Fields] OR "country s"[All Fields] OR "countrys"[All Fields])) OR (("middle"[All Fields] OR "middles"[All Fields]) AND ("income"[MeSH Terms] OR "income"[All Fields] OR "incomes"[All Fields]) AND ("countries"[All Fields] OR "country"[All Fields] OR "country s"[All Fields] OR "countrys"[All Fields])) OR ("developing countries"[MeSH Terms] OR ("developing"[All Fields] AND "countries"[All Fields]) OR "developing countries"[All Fields] OR ("developing"[All Fields] AND "country"[All Fields]) OR "developing country"[All Fields])
