# Supplementary material for: Understanding integrated service delivery: a scoping review of models for noncommunicable disease and mental health interventions in low-and-middle income countries
Source: BMC Health Serv Res. 2023 Jan 30;23:99. doi: 10.1186/s12913-023-09072-9 (PMC9885613; doi:10.1186/s12913-023-09072-9)
Supplement: Supplementary file 2 — Additional file 2: Appendix B. Delivery models identified at the community level. Appendix C. Delivery models identified at health centers. Appendix D. Delivery models identified at secondary level facilities. Appendix E. Delivery models identified at tertiary level institutions. Appendix F. Studies identified at specialty outpatient clinics. [file 12913_2023_9072_MOESM2_ESM.docx]

| **Appendix B. Delivery models identified at the community level** | | | | | | | | | | | | | | |
| --- | --- | --- | --- | --- | --- | --- | --- | --- | --- | --- | --- | --- | --- | --- |
| **Key** | **Urban/ rural** | **Scale** | **Institution** | **Research**  **role** | **Integration level** | **Mobile** | **Primary provider** | **Effort** | **Linkage** | **Task shifting** | **Decent- ralization** | **Compensation** | **Condition category** | **Services** |
| **Low HIV prevalence** | | | | | | | | | | | | | | |
| *Lower middle-income countries* | | | | | | | | | | | | | | |
| Rawal Bangladesh[1] | Mixed | Large scale | Public | Imbedded or evaluation | Description of existing delivery model | No | CHW | Part time | None | No | No | Not specified | Common chronic NCD, MCH | Health education, screening, medication dispensing |
| Nimgaonkar India A[2] | Rural | Single center | Public | Imbedded or evaluation | Description of existing delivery model | Yes | Midlevel provider | Not specified | Referral | No | Yes | Volunteer | Severe chronic NCD | Health education, screening, linkage |
| Chatterjee India[3] | Rural | Small to medium scale | Private, NGO | Pilot | New care delivery teams | No | CHW | Part time | Referral | No | Yes | Not specified | Severe NP | Health education, linkage, adherence support |
| Joshi India[4] | Rural | Large scale | Public | Pilot | New care delivery teams | No | CHW | Part time | Referral | No | No | Not specified | Common chronic NCD | Health education, screening, linkage, adherence support, medication dispensing, home visits, monitoring, medication management |
| Amarchand India A[5] | Not specified | Single center | Public | Pilot | New services integrated into existing delivery models | No | Layworker | NA | Referral | No | No | NA | Common chronic NCD, severe chronic NCD | Health education, screening, linkage, adherence support, home visits |
| Basu India[6] | Rural | Large scale | Public | Pilot | New care delivery teams | No | CHW | Full time | Referral | Yes | Yes | Salaried | Common chronic NCD, severe chronic NCD | Health education, screening, linkage |
| Abdel-All India[7] | Not specified | National | Public | Imbedded or evaluation | Description of existing delivery model | No | CHW | Not specified | Referral | No | No | Volunteer | Common chronic NCD, severe chronic NCD | Health promotion, health education, screening, linkage, adherence support, patient follow-up |
| Sahu India[8] | Not specified | Small to medium scale | Not specified | Pilot | Task redistribution within existing delivery models | No | CHW | Full time | Referral | Yes | No | Fee per service | Common chronic NCD, MCH, Sense organ | Screening |
| Rawal Nepal[9] | Mixed | Small to medium scale | Public | Imbedded or evaluation | Description of existing delivery model | No | CHW | Part time | Referral | Yes | Yes | Volunteer | Common chronic NCD, acute infection, MCH | Health promotion, health education, screening, linkage, initial diagnosis, adherence support, medication dispensing |
| Gyawali Nepal[10] | Peri-urban | Small to medium scale | Public | Protocol | New care delivery teams | No | CHW | Part time | Referral | No | Yes | Volunteer | Common chronic NCD | Health education, screening, linkage, adherence support, monitoring |
| Neupane Nepal[11] | Urban | Small to medium scale | NGO | Protocol | New care delivery teams | Yes | Multi-cadre team |  | Referral | No | Yes | Salaried | Common chronic NCD, severe chronic NCD | Health promotion, screening, initial diagnosis, medication dispensing, patient follow-up, monitoring |
| Welton-Mitchell Nepal[12] | Rural | Small to medium scale | NA | Pilot | New care delivery teams | No | Multi-cadre team | NA | No | No | No | NA | Common NP | Health education, peer-group facilitation |
| Ku Philippines B [13] | Mixed | Small to medium scale | Public | Experimental study | New care delivery teams | No | CHW | Not specified | No | Yes | No | Not specified | Common chronic NCD | Health education, adherence support, home visits |
| Fernando Sri Lanka [14] | Mixed | National | Public | Imbedded or evaluation | New care delivery teams | No | Multi-cadre team | Not specified | Referral | No | Yes | Not specified | Common NP | Health promotion, health education, screening, linkage, adherence support, medication dispensing, home visits, patient follow-up |
| Murphy Vietnam[15] | Rural | Large scale | Public | Protocol | New conditions and new services integrated into existing delivery models | No | Social worker | Not specified | Referral | No | No | Not specified | Common NP, PHC | Health education, screening, psychotherapy, patient follow-up |
| Nguyen Vietnam A[16] | Rural | Single center | Public | Pilot | New conditions integrated into existing delivery models | No | Not specified | NA | Not specified | No | No | NA | Common chronic NCD | Health promotion |
| Van Minh Vietnam A[17] | Rural | Small to medium scale | Public | Imbedded or evaluation | Description of existing delivery model | No | CHW | Not specified | Not specified | No | No | Stipend | Common chronic NCD | Health promotion, health education, screening |
| *Upper middle-income countries* | | | | | | | | | | | | | | |
| Falcone Brazil[18] | Urban | Single center | Not specified | Experimental study | New conditions and new services integrated into existing delivery models | No | Multidisciplinary team | NA | Referral | No | No | NA | Common NP, MCH | Health education, screening, linkage, peer-group facilitation |
| Struckmann Bulgaria[19] | Urban | Single center | NGO | Imbedded or evaluation | Description of existing delivery model | No | Multi-cadre team | NA | Referral | No | No | NA | Common chronic NCD | health promotion, health education, screening, linkage, acute care, psychotherapy, home visits, patient follow-up, medication management |
| Chen China 2020 [20] | Urban | Single center | NA | Pilot | Description of existing delivery model | No | Not specified | NA | No | No | No | NA | Severe NP | Health education |
| Zhong Chin[21]a | Urban | Small to medium scale | Public | Pilot | New care delivery teams | No | Lay worker | NA | Not specified | No | No | NA | Common chronic NCD | Health education, peer group facilitation |
| Kim Indonesia A [22] | Mixed | Small to medium scale | Public | Imbedded or evaluation | New conditions integrated into existing delivery models | Yes | Midlevel provider | Not specified | Referral | Yes | Yes | Not specified | Severe chronic NCD | Health promotion, screening, linkage, initial diagnosis |
| Tapia-Conyer Mexico A[23] | Not specified | Small to medium scale | Public | Imbedded or evaluation | New services integrated into existing delivery models | Yes | Multi-cadre team | NA | Referral | No | No | NA | Common chronic NCD | Health education, screening, linkage |
| Worster Mexico[24] | Rural | Small to medium scale | Public, NGO | Experimental study | New care delivery teams | No | CHW | Full time | Not specified | No | Yes | Salaried | Common chronic NCD | Health education, linkage, initial diagnosis, adherence support, home visits, patient follow-up, monitoring |
| Jiamjariyaporn Thailand B[25] | Rural | Small to medium scale | Public | Experimental study | New care delivery teams | No | CHW | Not specified | Not specified | No | No | Not specified | Common chronic NCD | Health education, adherence support, home visits, patient follow-up, monitoring |
| Turnacilar Turkey[26] | Urban | Small to medium scale | NA | Imbedded or evaluation | New services integrated into existing delivery models | No | Pharmacist | NA | No | No | No | NA | Common chronic NCD | Health education, adherence support, medication dispensing, patient follow-up, monitoring |
| *Mixed income* | | | | | | | | | | | | | | |
| Shields-Zeeman Multi [27] | Urban | Small to medium scale | Not specified | Protocol | New care delivery teams | No | Multi-cadre team | NA | Referral | Yes | Yes | NA | Common NP, severe NP | Linkage, psychotherapy, medication dispensing, home visits |
| **Medium HIV prevalence** | | | | | | | | | | | | | | |
| *Low-income countries* | | | | | | | | | | | | | | |
| Fekadu Ethiopia B[28] | Rural | Small to medium scale | Public | Feasibility study | New conditions integrated into existing delivery models | No | CHW | Full-time (HEW), part-time (HDA) | Referral | Yes | No | Salaried (HEW), volunteer (HDA) | Common NP, severe NP, acute infection, MCH | Health promotion, health education, case-finding, linkage, adherence support, acute care, home visits, patient follow-up, monitoring |
| Smith Rwanda B[29] | Rural | Small to medium scale | Public, NGO | Protocol | New conditions and new services integrated into existing delivery models | No | CHW | Not specified | Referral | Yes | Yes | Not specified | Common NP, severe NP | Health education, case-finding, adherence support, patient follow-up |
| *Lower middle-income countries* | | | | | | | | | | | | | | |
| Ngaruiya Kenya[30] | Urban | Small to medium scale | Not specified | Pilot | New care delivery teams | No | CHW | Not specified | Referral | Yes | Yes | Fee per service | Common chronic NCD | Health promotion, health education, screening, linkage, adherence support, peer-group facilitation |
| Pastakia Kenya[31] | Rural | Single center | Public, NGO | Feasibility study | New conditions integrated into existing delivery models | Yes | Multi-cadre team | NA | Referral | No | Yes | NA | Common chronic NCD, chronic infection | Health promotion, health education, screening, linkage |
| Rachlis Kenya 2016[32, 33] | Mixed | Small to medium scale | Public, NGO | Imbedded or evaluation | Description of existing delivery model | No | CHW | Not specified | Referral | No | No | Volunteer | Common chronic NCD, chronic infection | Health promotion, linkage, adherence support, home visits, monitoring |
| **High HIV prevalence** | | | | | | | | | | | | | | |
| *Low-income countries* | | | | | | | | | | | | | | |
| Angwenyi Malawi[34] | Rural | Single center | FBO | Pilot | Description of existing delivery model | No | CHW | Full time | Referral | No | No | Volunteer | Common chronic NCD, severe chronic NCD, common NP, chronic infection | Health promotion, linkage, adherence support, home visits, home-based care, monitoring |
| Kauye Malawi[35] | Not specified | Small to medium scale | Public | Pilot | New conditions integrated into existing delivery models | No | CHW | Part time | None | No | Not specified | Not specified | Common NP, chronic infection, acute infection, MCH | Health promotion, screening, case-finding, monitoring |
| Kachimanga Malawi B [36] | Rural | Small to medium scale | Public, NGO | Imbedded or evaluation | New conditions integrated into existing delivery models | Yes | Layworker | NA | Referral | Yes | No | NA | Common chronic NCD, severe chronic NCD, chronic infection | Screening, linkage |
| Banda Malawi B[37] | Not specified | Large scale | Private | Experimental study | New services integrated into existing delivery models | No | Informal providers | Not specified | Referral | No | No | NA | Common chronic NCD, chronic infection | Health education, case-finding, linkage, adherence support |
| Kwarisiima Uganda A[38] | Rural | Large scale | Public | Experimental study | New conditions integrated into existing delivery models | No | Not specified | Not specified | Referral | No | No | Not specified | Common chronic NCD, chronic infection | Health education, screening, linkage |
| Chamie Uganda[39] | Rural | Single center | Public | Feasibility study | New conditions integrated into existing delivery models | Yes | Counsellor | Part-time | Referral | No | No | Not specified | Common chronic NCD, chronic infection, acute infection | Health promotion, screening, linkage |
| Jack Uganda[40] | Mixed | Large scale | NGO | Imbedded or evaluation | New care delivery teams | No | CHW | Part-time | Referral | Yes | Yes | None | Severe chronic NCD | Health education, screening, linkage, medication dispensing, home visits, home-based care |
| *Lower middle-income countries* | | | | | | | | | | | | | | |
| Dunbar Malawi[41] | Rural | Large scale | Public, NGO | Pilot | New conditions integrated into existing delivery models | No | CHW | Full time | Referral | No | No | Salaried | Common chronic NCD, common NP, chronic infection, acute infection, MCH | Health promotion, health education, screening, case-finding, linkage, adherence support |
| Hartwig Tanzania[42] | Mixed | Large scale | FBO | Imbedded or evaluation | New care delivery teams | No | Multi-cadre team | Not specified | Referral | No | Yes | Volunteer | Severe chronic NCD | Linkage, medication dispensing, home visits |
| Duffy Zimbabwe A[43] | Mixed | Small to medium scale | Public, NGO | Pilot | New conditions integrated into existing delivery models | No | Multi-cadre team | Not specified | Referral | No | No | Not specified | Common NP, chronic infection | Health education, screening, linkage |
| *Upper middle-income countries* | | | | | | | | | | | | | | |
| Petersen South Africa B[44] | Rural | Small to medium scale | Public | Imbedded or evaluation | New conditions integrated into existing delivery models | No | CHW | Not specified | Not specified | No | No | Not specified | Common NP, PHC | Health promotion, adherence support, home visits, patient follow-up |
| Madela South Africa[45] | Mixed | Small to medium scale | Public, NGO | Imbedded or evaluation | New conditions integrated into existing delivery models | No | CHW | Not specified | Referral | No | Yes | Not specified | Common chronic NCD, chronic infection | Health education, screening, linkage monitoring |
| Schnippel South Africa[46] | Rural | Single center | NGO | Imbedded or evaluation | Description of existing delivery model | Yes | Midlevel provider | NA | Referral | No | No | NA | Severe chronic NCD, chronic infection, acute infection | Health education, screening, medication dispensing |
| O'Brien South Africa C[47] | Rural | Large scale | Public | Imbedded or evaluation | New care delivery teams | No | CHW | Not specified | Referral | No | No | Not specified | Severe chronic NCD | Linkage, home visits, home-based care |
| Ndou South Africa[48] | Not specified | Single center | Public, NGO | Pilot | New conditions integrated into existing delivery models | No | CHW | Part time | Referral | No | No | Not specified | Common chronic NCD, chronic infection, MCH | Health education, linkage, adherence support, medication dispensing, home visits |
| Govindasamy South Africa [49] | Mixed | Small to medium scale | Not specified | Imbedded or evaluation | New conditions integrated into existing delivery models | Yes | Midlevel provider | Not specified | Referral | No | Yes | Not specified | Common chronic NCD, chronic infection | Health promotion, screening, linkage |
| Müller South Africa[50] | Not specified | Single center | NA | Experimental study | New conditions and new services integrated into existing delivery models | No | Layworker | NA | Referral | No | No | NA | Common chronic NCD | Health promotion, health education, screening, linkage |
| Golovaty South Africa[51] | Not specified | Small to medium scale | Not specified | Imbedded or evaluation | New conditions integrated into existing delivery models | No | Counsellor | Not specified | Referral | No | No | Salaried | Common chronic NCD, common NP, chronic infection | Screening |
| Morris-Paxton South Africa[52] | Rural | Large scale | Public | Imbedded or evaluation | New care delivery teams | No | CHW | Full time | Referral | No | Yes | Not specified | Common chronic NCD, chronic infection, MCH | Screening, linkage |
| Botha South Africa[53] | Urban | Single center | Public | Experimental study | New services integrated into existing delivery models | Yes | Midlevel provider | NA | Referral | No | No | NA | Severe NP | Health promotion, linkage, adherence support, psychotherapy, medication dispensing, home visits, monitoring |
| Siedner South Africa[54] | Rural | Small to medium scale | Public | Imbedded or evaluation | New services integrated into existing delivery models | No | Not specified | NA | Referral | No | No | NA | Common chronic NCD | Screening, linkage, home visits |
| *Mixed income* | | | | | | | | | | | | | | |
| Kutcher Malawi Tanzania B[55] | Not specified | Multi-country | Public | Imbedded or evaluation | New conditions and new services integrated into existing delivery models | No | Layworker | NA | Referral | No | No | NA | Common NP | Health education, case-finding, linkage, peer-group facilitation |
| Guwatudde Uganda South Africa [56] | Mixed | Multi-country | Public | Experimental study | New conditions and new services integrated into existing delivery models | No | CHW | Not specified | Referral | Yes | No | Not specified | Common chronic NCD | Health promotion, screening, linkage, peer-group facilitation |

| **Appendix C: Delivery models identified at health centers** | | | | | | | | | | | |
| --- | --- | --- | --- | --- | --- | --- | --- | --- | --- | --- | --- |
| **Key** | **Urban/ rural** | **Scale** | **Institution** | **Research role** | **Integration level** | **Primary provider** | **Linkage** | **Task shifting** | **Decent- ralization** | **Condition category** | **Services** |
| **Low HIV prevalence** | | | | | | | | | | | |
| *Lower middle-income countries* | | | | | | | | | | | |
| Amarchand India B[5] | Not specified | Small to medium scale | Public | Pilot | New services integrated into existing delivery models | Midlevel provider | Referral | No | No | Common chronic NCD, severe chronic NCD | Health education, screening, linkage, initial diagnosis, medication dispensing, monitoring |
| Dorji Bhutan A[57] | Mixed | National | Public | Imbedded or evaluation | Description of existing delivery model | Midlevel provider | Not specified | No | Yes | Common chronic NCD | Health education, screening, initial diagnosis, medication dispensing, patient follow-up, monitoring, medication management |
| Khan Pakistan [58] | Not specified | Large scale | Public | Experimental study | New conditions integrated into existing delivery models | Generalist physician | Referral | No | No | Common chronic NCD, PHC | Health education, screening, linkage, initial diagnosis, medication dispensing, patient follow-up, monitoring |
| Khan Pakistan[59] | Rural | Large scale | Public | Experimental study | New conditions integrated into existing delivery models | Generalist physician | Not specified | No | No | Common chronic NCD, PHC | Health education, screening, initial diagnosis, adherence support, medication dispensing, patient follow-up, monitoring, medication management |
| Khan Pakistan[60] | Mixed | Small to medium scale | Public | Experimental study | New conditions integrated into existing delivery models | Generalist physician | Referral | No | No | Common chronic NCD, PHC | Health education, screening, linkage, initial diagnosis, medication dispensing, patient follow-up |
| Ku Philippines A[13] | Mixed | Small to medium scale | Public | Experimental study | New care delivery teams | Multi-cadre team | No | Yes | No | Common chronic NCD | Health promotion, health education, screening, case-finding, adherence support, home visits, patient follow-up, medication management |
| Lall India[61] | Rural | Small to medium scale | Public, private | Imbedded or evaluation | Description of existing delivery model | Generalist physician | Referral | No | No | Common chronic NCD | Health education, medication dispensing, monitoring |
| Lall India [62] | Rural | Small to medium scale | Public | Experimental study | Task redistribution within existing delivery models | Multi-cadre team | Counter-referral | Yes | No | Common chronic NCD | Screening, linkage, adherence support, medication dispensing, medication management |
| Mallawaarachchi Sri Lanka[63] | Mixed | National | Public | Imbedded or evaluation | Description of existing delivery model | Midlevel provider | Referral | No | No | Common chronic NCD | Health education, screening, linkage, initial diagnosis, Monitoring |
| Manjunatha India A[64] | Mixed | Large scale | Public | Imbedded or evaluation | New conditions integrated into existing delivery models | Midlevel provider | Referral | No | Yes | Common NP, PHC | Health education, linkage, medication dispensing |
| Murphy Vietnam[65] | Mixed | National | Public | Imbedded or evaluation | New conditions integrated into existing delivery models | Generalist physician | Referral | No | No | Common NP, PHC | Linkage |
| Nguyen Vietnam B[16] | Rural | Single center | Public | Pilot | New conditions integrated into existing delivery models | Not specified | Not specified | No | No | Common chronic NCD, PHC | Initial diagnosis, patient follow-up, monitoring, medication management |
| Piette Bolivia[66] | Urban | Single center | Public, private | Pilot | New services integrated into existing delivery models | Generalist physician | No | No | No | Common chronic NCD | Health education, adherence support |
| Spagnolo Tunisia[67] | Mixed | Small to medium scale | Public, private | Protocol | New conditions integrated into existing delivery models | Generalist physician | Referral | Yes | No | Common NP, severe NP, PHC | Initial diagnosis, medication management |
| Van Minh Vietnam B[17] | Rural | Small to medium scale | Public | Imbedded or evaluation | Description of existing delivery model | Not specified | Not specified | No | No | Common chronic NCD | Health education, screening, initial diagnosis, medication dispensing |
| Wangchuk Bhutan A[68] | Not specified | Small to medium scale | Public | Pilot | New conditions integrated into existing delivery models | Midlevel provider | Referral | Yes | No | Common chronic NCD, severe chronic NCD, PHC | Health education, screening, linkage, initial diagnosis, medication dispensing, home visits, patient follow-up, monitoring, medication management |
| *Upper middle-income countries* | | | | | | | | | | | |
| Anjara Indonesia[69] | Not specified | Small to medium scale | Public | Pilot | New conditions and new services integrated into existing delivery models | Generalist physician | Referral | Yes | No | Common NP, PHC | Linkage, initial diagnosis, psychotherapy, medication dispensing |
| Arevian Lebanon[70] | Urban | Single center | NGO | Imbedded or evaluation | New care delivery teams | Midlevel provider | Referral | No | No | Common chronic NCD | Health education, linkage, home visits, patient follow-up, monitoring, medication management |
| Barceló Mexico[71] | Urban | Large scale | Public | Imbedded or evaluation | New care delivery teams | Generalist physician | No | No | No | Common chronic NCD | Health education, linkage, patient follow-up, monitoring, medication management |
| Beganlic Bosnia and Herzegovina[72] | Urban | Single center | Public | Imbedded or evaluation | Description of existing delivery model | Generalist physician | Referral | No | No | Common chronic NCD | Health education, linkage, medication dispensing, patient follow-up, monitoring, medication management |
| Cerci Neto Brazil[73] | Urban | Small to medium scale | Public | Imbedded or evaluation | New care delivery teams | Generalist physician | Referral | No | No | Common chronic NCD | Health education, linkage, medication dispensing, home visits, patient follow-up |
| Chen China 2011[74] | Urban | Small to medium scale | Public | Protocol | New care delivery teams | Multi-cadre team | Referral | Yes | No | Common NP, PHC | Health education, screening, linkage, initial diagnosis, adherence support, medication dispensing, patient follow-up, monitoring |
| Chen China 2018[75] | Rural | Large scale | Public | Protocol | New care delivery teams | Generalist physician | Referral | Yes | No | Common chronic NCD | Health education, linkage, patient follow-up, monitoring |
| Chua Malaysia[76] | Urban | Small to medium scale | Private | Experimental study | New care delivery teams | Generalist physician | Not specified | Yes | No | Common chronic NCD | Health education, medication dispensing, patient follow-up |
| Cueto-Manzano Mexico[77] | Not specified | Single center | Public | Imbedded or evaluation | New conditions and new services integrated into existing delivery models | Multidisciplinary team | No | No | Yes | Common chronic NCD, PHC | Health promotion, health education, peer-group facilitation, patient follow-up |
| da Silva Marinho Brazil[78] | Urban | Small to medium scale | Public | Imbedded or evaluation | New services integrated into existing delivery models | Multi-cadre team | Referral | No | No | Common chronic NCD | Health education, screening, linkage, initial diagnosis, adherence support, home visits, patient follow-up, monitoring, medication management |
| Didier Brazil[79] | Peri-urban | Single center | Public | Experimental study | Task redistribution within existing delivery models | Multi-cadre team | No | Not specified | No | Common chronic NCD | Health education, adherence support, medication dispensing, patient follow-up, monitoring, medication management |
| Gagliardino Argentina[80] | Urban | Small to medium scale | Public | Imbedded or evaluation | New care delivery teams | Multidisciplinary team | Referral | No | No | Common chronic NCD | Health promotion, health education, linkage, medication dispensing, Patient follow-up, Monitoring, Medication management |
| Jiao China[81] | Urban | Large scale | Public | Imbedded or evaluation | New care delivery teams | Multidisciplinary team | No | No | No | Common chronic NCD | Health education, patient follow-up, medication management |
| Kim Indonesia B[22] | Mixed | Small to medium scale | Public | Imbedded or evaluation | New conditions integrated into existing delivery models | Generalist physician | Referral | No | Yes | PHC | Health education |
| Kuhmmer Brazil[82] | Not specified | Small to medium scale | Public | Imbedded or evaluation | New care delivery teams | Multidisciplinary team | Referral | No | No | Common chronic NCD | Health education, linkage, adherence support, medication dispensing |
| Li China [83] | Rural | Large scale | Public | Experimental study | New care delivery teams | Multi-cadre team | Not specified | No | Yes | Common chronic NCD, common NP | Health education, screening, initial diagnosis, adherence support, medication dispensing, home visits |
| Martins Brazil[84] | Urban | Small to medium scale | Public | Pilot | New care delivery teams | Generalist physician | Referral, counter-referral | No | No | Common chronic NCD | Screening, medication dispensing, patient follow-up, medication management |
| Miao China[85] | Rural | Small to medium scale | Public | Pilot | New care delivery teams | Generalist physician | Referral | Yes | No | Common chronic NCD | Health education, linkage, initial diagnosis, adherence support, medication dispensing, patient follow-up, monitoring, medication management |
| Mino-León Mexico[86] | Urban | Small to medium scale | Public | Experimental study | New services integrated into existing delivery models | Midlevel provider | Not specified | No | No | Common chronic NCD | Health education, adherence support, medication dispensing, home visits, patient follow-up |
| Pilipovic-Broceta Bosnia and Herzegovina[87] | Not specified | Small to medium scale | Not specified | Imbedded or evaluation | Description of existing delivery model | Generalist physician | No | No | No | Common chronic NCD | Health education, medication dispensing, patient follow-up, monitoring, medication management |
| Prestes Argentina[88] | Peri-urban | Single center | Public | Experimental study | Not specified | Multi-cadre team | Not specified | No | No | Common chronic NCD | Health education, screening, initial diagnosis, medication dispensing, patient follow-up, monitoring |
| Ramli Malaysia[89] | Urban | Large scale | Public | Experimental study | New care delivery teams | Generalist physician | No | No | No | Common chronic NCD | Health education, patient follow-up, monitoring, medication management |
| Sharifi Iran A[90] | Urban | Small to medium scale | Public, private | Experimental study | New services integrated into existing delivery models | Generalist physician | Referral | Yes | Yes | Common NP, severe NP | Health education, screening, linkage, initial diagnosis, patient follow-up, medication management |
| Shi China A[91] | Rural | Small to medium scale | Public | Imbedded or evaluation | New services integrated into existing delivery models | Generalist physician | Referral, counter-referral | No | No | Common chronic NCD | Health education, linkage, initial diagnosis, patient follow-up, medication management |
| Tapia-Conyer Mexico B[23] | Not specified | Small to medium scale | Public | Imbedded or evaluation | New services integrated into existing delivery models | Multi-cadre team | Referral | No | No | Common chronic NCD | Initial diagnosis |
| Torrey Colombia[92] | Mixed | Small to medium scale | Public | Experimental study | New conditions and new services integrated into existing delivery models | Generalist physician | None | No | No | Common NP, PHC | Screening, initial diagnosis, psychotherapy |
| Tu China B[93] | Urban | Small to medium scale | Public | Imbedded or evaluation | New services integrated into existing delivery models | Multi-cadre team | Referral | Yes | No | Common chronic NCD | health education, linkage, medication dispensing, patient follow-up, monitoring |
| Wesseling Costa Rica[94] | Mixed | Small to medium scale | Public | Feasibility study | New conditions integrated into existing delivery models | Multi-cadre team | Not specified | No | Yes | Severe NP, PHC | Screening, initial diagnosis |
| Wong China[95] | Urban | Small to medium scale | Public | Imbedded | New services integrated into existing delivery models | Not specified | Not specified | No | No | Common chronic NCD, PHC | Health education, patient follow-up |
| Xu China[96] | Urban | Small to medium scale | Public | Protocol | New care delivery teams | Generalist physician | Referral | No | No | Common chronic NCD | Health education, linkage, adherence support, patient follow-up, monitoring, medication management |
| Yin China[97] | Urban | National | Public | Imbedded or evaluation | New care delivery teams | Generalist physician | Referral | No | No | Common chronic NCD, common NP | Health education, linkage, initial diagnosis, medication dispensing, patient follow-up |
| Zhu China[98] | Urban | Single center | Public | Pilot | New services integrated into existing delivery models | Midlevel provider | Referral | Yes | No | Common chronic NCD | Health education, linkage, medication dispensing, home visits, monitoring, medication management |
| **Medium HIV prevalence** | | | | | | | | | | | |
| *Low-income countries* | | | | | | | | | | | |
| Fekadu Ethiopia A [28] | Rural | Small to medium scale | Public, NGO | Imbedded or evaluation | New conditions and new services integrated into existing delivery models | Midlevel provider | Referral, counter-referral | No | Yes | Common chronic NCD, common NP, PHC | Linkage, initial diagnosis, medication dispensing, patient follow-up, medication management |
| Labhardt Cameroon[99] | Rural | Small to medium scale | Public, NGO | Imbedded or evaluation | New conditions integrated into existing delivery models | Midlevel provider | Referral | Yes | No | Common chronic NCD, PHC | Health education, linkage, initial diagnosis, medication dispensing |
| Mamo Ethiopia[100] | Rural | Small to medium scale | Public, NGO | Imbedded or evaluation | New conditions and new services integrated into existing delivery models | Midlevel provider | Referral, counter-referral | No | Yes | Common chronic NCD, common NP, PHC | Linkage, initial diagnosis, medication dispensing, patient follow-up, medication management |
| Ndayisaba Rwanda[101] | Rural | Small to medium scale | Public, NGO | Imbedded or evaluation | New services integrated into existing delivery models | Midlevel provider | Referral, counter-referral | Yes | Yes | Common chronic NCD | Screening, linkage, initial diagnosis, medication dispensing, monitoring |
| Smith Rwanda A[29] | Rural | Small to medium scale | Public, NGO | Protocol | New conditions integrated into existing delivery models | Midlevel provider | Referral | Yes | Yes | Common NP, severe NP, PHC | Health education, linkage, initial diagnosis, patient follow-up |
| Zou Sierra Leone[102] | Mixed | Small to medium scale | Public, NGO | Feasibility study | New services integrated into existing delivery models | Midlevel provider | Referral | No | No | Common chronic NCD | Health education, linkage, initial diagnosis, medication dispensing, patient follow-up |
| *Lower middle-income countries* | | | | | | | | | | | |
| Adewuya Nigeria[103] | Urban | Small to medium scale | Public | Feasibility study | New conditions and new services integrated into existing delivery models |  | Referral | Yes | No | Common NP, PHC | Health education, screening, linkage, initial diagnosis, adherence support, psychotherapy, medication dispensing, patient follow-up |
| Adler Ghana[104] | Peri-urban | Small to medium scale | Public | Imbedded or evaluation | New conditions integrated into existing delivery models | Midlevel provider | Referral, counter-referral | Yes | No | Common chronic NCD, chronic infection, acute infection, MCH | Health education, screening, linkage, initial diagnosis, adherence support, medication dispensing, monitoring, medication management |
| Claeys Kenya[105] | Mixed | Small to medium scale | NGO | Imbedded or evaluation | Description of existing delivery model | Midlevel provider | Referral | No | No | Severe chronic NCD | Health education, screening, linkage |
| Gureje Ghana Nigeria[106] | Mixed | Multi-country | Public, private | Protocol | New care delivery teams | Complementary alternative health provider | Referral | Yes | No | Severe NP | Linkage, medication dispensing, patient follow-up, monitoring |
| Gureje Nigeria[107] | Mixed | Large scale | Public | Pilot | New conditions integrated into existing delivery models | Midlevel provider | Referral | Yes | Yes | Common NP, Severe NP, PHC | Linkage, initial diagnosis, medication dispensing |
| Huchko Kenya A[108] | Not specified | Small to medium scale | Public, NGO | Pilot | New conditions integrated into existing delivery models | Midlevel provider | Not specified | No | No | Severe chronic NCD, chronic infection | Health education, screening |
| Jenkins Kenya[109] | Mixed | National | Public | Imbedded or evaluation | New conditions integrated into existing delivery models | Generalist physician | Not specified | No | No | Common NP, PHC | Initial diagnosis, medication dispensing, patient follow-up |
| Kengne Cameroon[110] | Rural | Small to medium scale | Not specified | Pilot | New care delivery teams | Midlevel provider | Not specified | Yes | No | Common chronic NCD, common NP, chronic infection | Health education, screening, linkage, initial diagnosis, acute care, medication dispensing, patient follow-up |
| Khabala Kenya[111] | Urban | Single center | Public, NGO | Imbedded or evaluation | New services integrated into existing delivery models | Midlevel provider | Referral | No | No | Common chronic NCD, chronic infection, MCH | Health promotion, linkage, adherence support, peer-group facilitation, medication dispensing, monitoring |
| Kumar Kenya[112] | Urban | Small to medium scale | Public | Protocol | New conditions and new services integrated into existing delivery models | Multi-cadre team | Referral | No | Yes | Common NP, PHC, MCH | Health education, screening, linkage, psychotherapy, peer-group facilitation |
| Nnodu Nigeria[113] | Not specified | Small to medium scale | Public | Feasibility study | New conditions and new services integrated into existing delivery models | Midlevel provider | No | No | Yes | Severe chronic NCD, acute infection | Health education, screening, adherence support, medication dispensing, patient follow-up |
| Osetinsky Kenya[114] | Not specified | Large scale | Public, NGO | Experimental study | New conditions integrated into existing delivery models | Midlevel provider | Referral | No | No | Common chronic NCD, common NP, chronic infection, MCH | Initial diagnosis, adherence support, medication dispensing, monitoring |
| Some Kenya[115] | Urban | Small to medium scale | Public, NGO | Imbedded or evaluation | Task redistribution within existing delivery models | Midlevel provider | Not specified | Yes | No | Common chronic NCD, severe chronic NCD, common NP, chronic infection, MCH | Initial diagnosis, acute care, medication dispensing, monitoring |
| **High HIV prevalence** | | | | | | | | | | | |
| *Low-income countries* | | | | | | | | | | | |
| Banda Malawi A[37] | Not specified | Large scale | Public | Experimental study | New services integrated into existing delivery models | Midlevel provider | Referral | No | No | Common chronic NCD, chronic infection | Health promotion, health education, screening, linkage, initial diagnosis, medication dispensing |
| Gutnik Malawi[116] | Urban | Small to medium scale | Public | Feasibility study | New services integrated into existing delivery models | CHW | Referral | Yes | No | Severe chronic NCD | Health education, screening, linkage |
| Kwarisiima Uganda[38] | Not specified | Large scale | Public | Experimental study | New conditions integrated into existing delivery models | Not specified | Referral | No | No | Common chronic NCD, chronic infection | Linkage, initial diagnosis, medication dispensing, patient follow-up, monitoring, medication management |
| Muddu Uganda[117] | Not specified | Small to medium scale | Public | Feasibility study | New services integrated into existing delivery models | CHW | Referral | Yes | No | Severe chronic NCD | Health education, screening, linkage |
| Wroe Malawi[118] | Rural | Large scale | Public, NGO | Imbedded or evaluation | New services integrated into existing delivery models | Midlevel provider | Referral, counter-referral | No | Yes | Common chronic NCD, common NP, severe NP, chronic infection | Screening, linkage, adherence support, psychotherapy, medication dispensing, patient follow-up, monitoring, medication management |
| *Lower middle-income countries* | | | | | | | | | | | |
| Mwanahamuntu Zambia[119] | Urban | Small to medium scale | Public, bilateral-supported | Pilot | New conditions integrated into existing delivery models | Midlevel provider | Referral | Yes | No | Severe chronic NCD, chronic infection | Health promotion, health education, screening, linkage |
| Chibanda Zimbabwe[120] | Urban | Small to medium scale | Public | Pilot | New conditions and new services integrated into existing delivery models | Lay worker | Referral | Yes | Yes | Common NP, PHC | Linkage, psychotherapy, peer group facilitation, home visits, patient follow-up |
| Duffy Zimbabwe[43] | Mixed | Large scale | Public, NGO | Imbedded or evaluation | New conditions integrated into existing delivery models | Midlevel provider | Referral | Yes | Yes | Common chronic NCD, chronic infection, PHC | Health education, screening, initial diagnosis, medication dispensing, patient follow-up, monitoring |
| Frieden Zimbabwe[121] | Rural | Small to medium scale | Public, NGO | Pilot | New conditions and new services integrated into existing delivery models | Midlevel provider | Referral, counter-referral | No | No | Common NP, chronic infection | Health education, screening, linkage, psychotherapy |
| *Upper middle-income countries* | | | | | | | | | | | |
| Ameh South Africa[122] | Not specified | Large scale | Public | Pilot | New conditions integrated into existing delivery models | Midlevel provider | Not specified | Yes | No | Common chronic NCD, common NP, chronic infection | Linkage, medication dispensing, patient follow-up |
| Coleman South Africa[123] | Mixed | Small to medium scale | Public | Imbedded or evaluation | New services integrated into existing delivery models | Multi-cadre team | Referral, counter-referral | Yes | No | Common chronic NCD, common NP, PHC | Linkage, initial diagnosis, patient follow-up, monitoring, medication management , medication management |
| Fairall South Africa[124] | Rural | Large scale | Public | Pilot | New conditions and new services integrated into existing delivery models | Midlevel provider | Referral | No | No | Common NP, chronic infection | Health education, screening, initial diagnosis, medication dispensing, patient follow-up |
| Hlongwa South Africa[125] | Mixed | Small to medium scale | Public | Imbedded or evaluation | New conditions integrated into existing delivery models | Midlevel provider | Referral | Yes | No | Common NP, PHC | Screening, linkage, medication management |
| Khan South Africa[126] | Urban | Small to medium scale | Public | Imbedded or evaluation | New services integrated into existing delivery models | Multi-cadre team | Referral | Yes | No | Severe chronic NCD | Screening, linkage |
| Lebina South Africa [127] | Not specified | Large scale | Public | Pilot | New services integrated into existing delivery models | Multi-cadre team | Counter-referral | No | No | Common chronic NCD, common NP, chronic infection | Linkage, adherence support, medication dispensing, home visits,  , medication management |
| Lovero South Africa[128] | Mixed | Small to medium scale | Public | Imbedded or evaluation | New conditions and new services integrated into existing delivery models | Multidisciplinary team | Referral | No | Yes | Common NP, chronic infection, MCH | Screening, linkage, initial diagnosis, psychotherapy, medication dispensing |
| Lund South Africa[129] | Urban | Small to medium scale | Public | Protocol | New conditions and new services integrated into existing delivery models | CHW | Referral | Yes | No | Common NP, MCH | Health education, screening, linkage, psychotherapy, patient follow-up |
| Maconick South Africa[130] | Rural | Single center | Public | Experimental study | Task redistribution within existing delivery models | Midlevel provider | Referral | Yes | No | Common chronic NCD, common NP, Severe NP | Screening, linkage |
| Mahomed South Africa[131] | Not specified | Large scale | Public | Imbedded or evaluation | Task redistribution within existing delivery models | Midlevel provider | Counter-referral | No | No | Chronic infection | Medication management |
| Myers South Africa[132] | Mixed | Large scale | Public | Protocol | New conditions and new services integrated into existing delivery models | CHW | Referral | No | No | Common NP, chronic infection, PHC | Health education, linkage, psychotherapy |
| Petersen South Africa A[44] | Rural | Large scale | Public | Protocol | New conditions and new services integrated into existing delivery models | CHW | Referral | No | No | Common NP, chronic infection, PHC | Health education, linkage, psychotherapy |
| Spedding South Africa[133] | Not specified | Single center | Public | Feasibility study | New conditions and new services integrated into existing delivery models | Midlevel provider | Referral | Yes | No | Common NP, MCH | Screening, linkage, initial diagnosis, psychotherapy |
| Ramogola-Masire Botswana[134] | Urban | Small to medium scale | Not specified | Pilot | New conditions integrated into existing delivery models | Midlevel provider | Referral | No | No | Severe chronic NCD, chronic infection | Health education, screening, linkage |
| Thorogood South Africa[135] | Rural | Small to medium scale | Public | Protocol | Task redistribution within existing delivery models | Multi-cadre team | None | Yes | No | Common chronic NCD, chronic infection | Initial diagnosis, adherence support, medication dispensing, patient follow-up, medication management |
| *Mixed income* | | | | | | | | | | | |
| Kutcher Malawi Tanzania C[55] | Not specified | Multi-country | Public | Imbedded or evaluation | New conditions integrated into existing delivery models | Midlevel provider | No | No | No | Common NP | Health education, screening, initial diagnosis, psychotherapy |
| Van Hout Tanzania Uganda [136] | Urban | Large scale | Public | Protocol | New care delivery teams | Midlevel provider | Not specified | No | No | Common chronic NCD, chronic infection | Health education, linkage, initial diagnosis, medication dispensing, monitoring, medication management |
| Guwatudde Uganda South Africa A [56] | Mixed | Multi-country | Public | Experimental study | New services integrated into existing delivery models | Midlevel provider | Not specified | Yes | No | Common chronic NCD | Health education, medication dispensing, patient follow-up, monitoring, medication management |

| **Appendix D: Delivery models identified at secondary level facilities** | | | | | | | | | | | |
| --- | --- | --- | --- | --- | --- | --- | --- | --- | --- | --- | --- |
| **Key** | **Urban/ rural** | **Scale** | **Institution** | **Research role** | **Integration level** | **Primary provider** | **Linkage** | **Task shifting** | **Decent- ralization** | **Condition category** | **Services** |
| **Low HIV prevalence** | | | | | | | | | | | |
| *Low-income countries* | | | | | | | | | | | |
| Ansbro Democratic Republic of Congo[137] | Rural | Single center | Public, NGO | Imbedded or evaluation | New care delivery teams | Midlevel provider | Not specified | Yes | No | Common chronic NCD, severe chronic NCD | Health promotion, health education, initial diagnosis, peer-group facilitation, medication dispensing, patient follow-up, monitoring, medication management , medication management |
| *Lower middle-income countries* | | | | | | | | | | | |
| Huque Bangladesh[138] | Urban | Small to medium scale | Public | Feasibility study | New conditions integrated into existing delivery models | Generalist physician | Referral | No | No | Common chronic NCD, PHC | Health education, linkage, initial diagnosis, medication dispensing, patient follow-up, medication management |
| Wangchuk Bhutan B[68] | Not specified | Small to medium scale | Public | Pilot | New conditions integrated into existing delivery models | Midlevel provider | Referral | Yes | Yes | Common chronic NCD, severe chronic NCD, PHC | Health education, screening, linkage, initial diagnosis, medication dispensing, home visits, patient follow-up, monitoring, medication management , medication management |
| Dorji Bhutan B[57] | Mixed | National | Public | Imbedded or evaluation | Description of existing delivery model | Midlevel provider | Not specified | No | Yes | Common chronic NCD | Health education, screening, initial diagnosis, medication dispensing, patient follow-up, monitoring, medication management |
| Nimgaonkar India B[2] | Rural | Single center | Public | Imbedded or evaluation | Description of existing delivery model | Midlevel provider | Referral | No | Yes | Severe chronic NCD | Health education, linkage, initial diagnosis, medication dispensing, patient follow-up |
| Shukla India[139] | Not specified | Small to medium scale | Public | Pilot | New services integrated into existing delivery models | Midlevel provider | Referral | Yes | No | Common chronic NCD | Health promotion, screening, linkage |
| Manjunatha India B[64] | Mixed | Large scale | Public | Imbedded or evaluation | New conditions integrated into existing delivery models | Midlevel provider | Referral | No | Yes | Common NP | Health education, linkage, medication dispensing |
| Srinivasapura Venkateshmurthy India[140] | Mixed | Small to medium scale | Public | Protocol | Task redistribution within existing delivery models | Midlevel provider | Referral | Yes | No | Common chronic NCD | Health education, initial diagnosis, adherence support, medication dispensing, patient follow-up, monitoring |
| Collins Moldova[141] | Mixed | Small to medium scale | Public | Experimental study | New conditions integrated into existing delivery models | Generalist physician | None | No | No | Common chronic NCD, severe chronic NCD, PHC | Health education, initial diagnosis, medication dispensing, medication management , medication management |
| Van Minh Vietnam C[17] | Rural | Small to medium scale | Public | Imbedded or evaluation | Description of existing delivery model | Generalist physician | Not specified | No | No | Common chronic NCD | Health education, initial diagnosis, medication dispensing |
| *Upper middle-income countries* | | | | | | | | | | | |
| Mourao Brazil[142] | Urban | Single center | Public | Imbedded or evaluation | New care delivery teams | Specialist physician | None | No | No | Severe chronic NCD | Health education, adherence support, medication dispensing, patient follow-up |
| Li China 2016[143] | Not specified | National | Public | Imbedded or evaluation | New care delivery teams | Generalist physician | Not specified | No | No | Common chronic NCD | Health education, medication dispensing, patient follow-up, monitoring |
| Shi China B[91] | Rural | Small to medium scale | Public | Imbedded or evaluation | Task redistribution within existing delivery models | Generalist physician | Counter-referral | Yes | No | Common chronic NCD | Linkage, patient follow-up, medication management , medication management |
| Chao China[144] | Urban | Small to medium scale | Public | Pilot | New services integrated into existing delivery models | Not specified | No | No | No | Common chronic NCD | Health education, psychotherapy, patient follow-up, monitoring, medication management |
| Chen China 2018[145] | Urban | Single center | Public | Pilot | New services integrated into existing delivery models | Multi-cadre team | Referral, counter-referral | No | No | Common chronic NCD | Linkage, initial diagnosis, medication dispensing, patient follow-up, monitoring |
| Silva-Tinoco Mexico[146] | Urban | Large scale | Public | Imbedded or evaluation | New care delivery teams | Multidisciplinary team | None | No | No | Common chronic NCD | Health education, medication dispensing, patient follow-up, monitoring |
| Jiamjariyaporn Thailand A[25] | Rural | Small to medium scale | Public | Experimental study | New care delivery teams | Multidisciplinary team | Not specified | No | No | Common chronic NCD | Health education, medication dispensing, patient follow-up, monitoring, medication management |
| **Medium HIV Prevalence** | | | | | | | | | | | |
| *Lower-income countries* | | | | | | | | | | | |
| Walsh Haiti[147] | Urban | Single center | Not specified | Imbedded or evaluation | New conditions integrated into existing delivery models | Generalist physician | Referral | No | No | Common chronic NCD, chronic infection | Health education, screening, linkage, initial diagnosis, medication dispensing, patient follow-up |
| Rusingiza Rwanda[148] | Rural | Small to medium scale | Public, NGO | Imbedded or evaluation | New conditions and services integrated into existing delivery models | Midlevel provider | Referral, counter-referral | Yes | Yes | Severe chronic NCD | Linkage, initial diagnosis, medication dispensing, patient follow-up |
| Eberly Rwanda[149] | Rural | Single center | Public, NGO | Imbedded or evaluation | New conditions and new services integrated into existing delivery models | Midlevel provider | Referral, counter-referral | Yes | No | Common chronic NCD, severe chronic NCD | Initial diagnosis, medication dispensing, patient follow-up, medication management , medication management |
| Eberly Rwanda 2018[150] | Rural | Small to medium scale | Public, NGO | Imbedded or evaluation | New conditions and new services integrated into existing delivery models | Midlevel provider | Referral | Yes | Yes | Severe chronic NCD | Linkage, initial diagnosis, medication dispensing, patient follow-up, monitoring, medication management |
| Rutayisire Rwanda [151] | Rural | Single center | Public, NGO | Imbedded or evaluation | New conditions integrated into existing delivery models | Midlevel provider | Referral, counter-referral | Yes | No | Common chronic NCD, severe chronic NCD | Initial diagnosis, adherence support, medication dispensing, monitoring, medication management , medication management |
| *Lower middle-income countries* | | | | | | | | | | | |
| Huchko Kenya B[108] | Not specified | Small to medium scale | Public, NGO | Pilot | New conditions integrated into existing delivery models | Midlevel provider | Not specified | No | No | Severe chronic NCD, chronic infection | Health education, screening |
| Osetinsky Kenya[114] | Not specified | Small to medium scale | Public, NGO | Imbedded or evaluation | New conditions integrated into existing delivery models | Midlevel provider | Referral | No | No | Common chronic NCD, severe chronic NCD, common NP, chronic infection, MCH | Health education, initial diagnosis, medication dispensing, monitoring, medication management , medication management |
| Levy Kenya[152] | Mixed | Single center | Public | Protocol | New care delivery teams | Multi-cadre team | No | Yes | No | Common NP, severe NP | Psychotherapy, medication dispensing, monitoring |
| Odafe Nigeria[153] | Urban | Single center | Public, NGO | Imbedded or evaluation | New conditions integrated into existing delivery models | Midlevel provider | Referral | No | No | Severe chronic NCD, chronic infection, MCH | Health education, screening, linkage |
| **High HIV prevalence** | | | | | | | | | | | |
| *Lower-income countries* | | | | | | | | | | | |
| Gaynes Malawi[154] | Not specified | Single center | Public | Pilot | New conditions and new services integrated into existing delivery models | Multi-cadre team | No | No | No | Common NP | Screening, initial diagnosis, psychotherapy, medication dispensing, patient follow-up, monitoring |
| Talama Malawi[155] | Rural | Single center | Public, NGO | Imbedded or evaluation | New conditions and new services integrated into existing delivery models | Midlevel provider | Referral, counter-referral | No | No | Common chronic NCD, severe chronic NCD, common NP, chronic infection, MCH | Health promotion, health education, screening, linkage, initial diagnosis, adherence support, medication dispensing, monitoring, medication management , medication management |
| Kachimanga Malawi A[36] | Rural | Single center | Public, NGO | Imbedded or evaluation | New conditions integrated into existing delivery models | Layworker | Referral | Yes | No | Common chronic NCD, severe chronic NCD, chronic infection, PHC | Screening, linkage |
| *Lower middle-income countries* | | | | | | | | | | | |
| Frieden Zimbabwe B[121] | Rural | Large scale | Public, NGO | Imbedded or evaluation | New conditions integrated into existing delivery models | Generalist physician | Referral | Yes | Yes | Common chronic NCD, chronic infection | Health education, screening, initial diagnosis, medication dispensing, patient follow-up, monitoring |
| *Upper middle-income countries* | | | | | | | | | | | |
| O'Brien South Africa B[47] | Rural | Large scale | Public | Imbedded or evaluation | New care delivery teams | Multi-cadre team | Referral, counter-referral | No | No | Severe chronic NCD | Linkage |

| **Appendix E: Delivery models identified at tertiary level institutions** | | | | | | | | | | | |
| --- | --- | --- | --- | --- | --- | --- | --- | --- | --- | --- | --- |
| **Key** | **Urban/ rural** | **Scale** | **Institution** | **Research Role** | **Integration level** | **Primary provider** | **Linkage** | **Task shifting** | **Decent- ralization** | **Condition category** | **Services** |
| **Low HIV prevalence** | | | | | | | | | | | |
| *Lower middle-income countries* | | | | | | | | | | | |
| Ali India B[156] | Urban | Small to medium scale | Public | Experimental study | New care delivery teams | Multidisciplinary team | No | No | No | Common chronic NCD, common NP | Health education, screening, adherence support, psychotherapy, medication dispensing, patient follow-up, monitoring, medication management |
| Amarchand India C[5] | Not specified | Small to medium scale | Public | Pilot | New services integrated into existing delivery models | Multi-cadre team | No | No | No | Common chronic NCD, severe chronic NCD | Health education, screening, initial diagnosis, medication dispensing, monitoring |
| Atif Pakistan[157] | Mixed | Single center | Public | Pilot | New conditions and new services integrated into existing delivery models | Midlevel provider | No | No | No | Common NP, MCH | Psychotherapy |
| Dorji Bhutan C[57] | Mixed | National | Public | Imbedded | Description of existing delivery model | Generalist physician | Not specified | No | No | Common chronic NCD | Health education, screening, initial diagnosis, medication dispensing, patient follow-up, monitoring, medication management |
| Janssens Cambodia[158] | Urban | Small to medium scale | Public, NGO | Pilot | New care delivery teams | Generalist physician | No | No | No | Common chronic NCD, chronic infection | Health education, initial diagnosis, adherence support, peer group facilitation, patient follow-up, medication management |
| *Upper middle-income countries* | | | | | | | | | | | |
| Aguiar Brazil[159] | Urban | Single center | Public | Experimental study | Task redistribution within existing delivery models | Pharmacist | No | Yes | No | Common chronic NCD | Health education, linkage, adherence support, patient follow-up, monitoring, medication management |
| Aydin Turkey[160] | Urban | Single center | Public | Imbedded | Description of existing delivery model | Multidisciplinary team | No | No | No | Common chronic NCD | Acute care, medication dispensing |
| Benzecry Brazil [161] | Not specified | Single center | Not specified | Experimental study | New care delivery teams | Multidisciplinary team | Not specified | No | No | Severe chronic NCD | Health education, patient follow-up, monitoring |
| Chung Malaysia[162] | Not specified | Small to medium scale | Not specified | Pilot | New services integrated into existing delivery models | Pharmacist | No | No | No | Common chronic NCD | Health education, adherence support, patient follow-up, monitoring, medication management |
| El-Khoury Lebanon[163] | Urban | Single center | Not specified | Imbedded | Task redistribution within existing delivery models | Generalist physician | No | Yes | No | Severe NP | Health promotion, health education, psychotherapy, medication dispensing, home visits, monitoring |
| Li China 2018[164] | Not specified | Large scale | Not specified | Experimental study | New conditions and new services integrated into existing delivery models | Multi-cadre team | Referral | Yes | No | Severe chronic NCD, common NP | Health education, screening, linkage, initial diagnosis, psychotherapy, medication dispensing, patient follow-up, monitoring, medication management |
| Lim Malaysia[165] | Urban | Single center | Public | Experimental study | New services integrated into existing delivery models | Generalist physician | Not specified | No | No | Common chronic NCD | Health education, adherence support, medication dispensing, monitoring, medication management |
| Pinotti Brazil [166] | Urban | Single center | Not specified | Imbedded or evaluation | Description of existing delivery model | Generalist physician | No | No | No | Common chronic NCD, severe chronic NCD, chronic infection, acute infection, MCH | Health education, screening, initial diagnosis, medication dispensing, patient follow-up |
| Tu China A[93] | Urban | Small to medium scale | Public | Imbedded | New services integrated into existing delivery models | Multi-cadre team | Referral, counter-referral | Yes | No | Common chronic NCD | Health education, linkage, medication dispensing, patient follow-up, medication management |
| Tutino China[167] | Not specified | Large scale | Not specified | Experimental study | New services integrated into existing delivery models | Midlevel provider | No | No | No | Common chronic NCD | Health education, adherence support, patient follow-up |
| Yesil Turkey[168] | Urban | Single center | Not specified | Imbedded | New care delivery teams | Multi-cadre team | Not specified | No | No | Common chronic NCD | Health education, acute care, medication dispensing, patient follow-up |
| Zheng China[169] | Urban | Single center | Public | Pilot | New care delivery teams | Multidisciplinary team | No | No | No | Common NP | Health education, screening, linkage, initial diagnosis, adherence support, medication dispensing, patient follow-up |
| **Medium HIV Prevalence** | | | | | | | | | | | |
| *Lower-income countries* | | | | | | | | | | | |
| Jerene Ethiopia[170] | Rural | Small to medium scale | Public | Feasibility study | New conditions integrated into existing delivery models | Midlevel provider | Referral | No | No | Common chronic NCD, chronic infection | Screening, linkage |
| *Lower middle-income countries* | | | | | | | | | | | |
| Vodicka Kenya [171] | Urban | Small to medium scale | Public, FBO | Imbedded | New conditions integrated into existing delivery models | Midlevel provider | Not specified | No | No | Severe chronic NCD, chronic infection | Screening |
| Were Kenya[172] | Urban | Single center | Pilot | Pilot | New conditions and new services integrated into existing delivery models | Midlevel provider | Referral | No | No | Severe chronic NCD, acute infection, MCH | Screening, linkage |
| **High HIV prevalence** | | | | | | | | | | | |
| *Low-income countries* | | | | | | | | | | | |
| Brown Malawi[173] | Urban | Small to medium | Public | Imbedded | New care delivery teams | Multidisciplinary team | No | No | No | Severe chronic NCD | Medication dispensing |
| Pfaff Malawi[174] | Urban | Single center | Public, NGO-supported | Imbedded | New conditions integrated into existing delivery models | Midlevel provider | Referral | No | No | Severe chronic NCD, chronic infection | Screening, linkage, initial diagnosis, acute care, medication dispensing, patient follow-up |
| *Lower middle-income countries* | | | | | | | | | | | |
| Adams Tanzania[175] | Not specified | Single center | Public | Feasibility study | New conditions integrated into existing delivery | Midlevel provider | Referral | Yes | No | Common NP, chronic infection | Linkage, patient follow-up, monitoring, medication management |
| Palma eSwatini[176] | Urban | Single center | Public, bilateral-supported | Pilot | New conditions integrated into existing delivery models | Multi-cadre team | Referral | No | No | Common chronic NCD, chronic infection | Health education, screening, linkage |
| Rabkin eSwatini[177] | Urban | Single center | Public | Feasibility study | New conditions integrated into existing delivery models | Midlevel provider | Referral | No | No | Common chronic NCD, chronic infection | Health education, screening, linkage |
| *Upper middle-income countries* | | | | | | | | | | | |
| Hopkins South Africa[178] | Urban | Single center | Public | Imbedded | New conditions integrated into existing delivery models | Counsellor | Referral | No | No | Common chronic NCD, severe chronic NCD, chronic infection, acute infection | Health education, screening, linkage |
| Jonsson South Africa[179] | Urban | Single center | Public | Imbedded | Description of existing delivery model | Specialist physician | No | No | No | Common NP, chronic infection | Health education, screening, initial diagnosis, adherence support, psychotherapy, peer-group facilitation, medication dispensing, home visits, patient follow-up, monitoring |
| Kajee South Africa[180] | Urban | Single center | Public | Imbedded | New care delivery teams | Multidisciplinary team | No | No | No | Severe chronic NCD | Health education, medication dispensing, monitoring, medication management |
| O’Brien South Africa A[47] | Rural | Large scale | Public | Imbedded | New care delivery teams | Multidisciplinary team | Counter-referral | No | No | Severe chronic NCD | Linkage, patient follow-up |
| Pillay South Africa[181] | Urban | Single center | Public | Imbedded | New care delivery teams | Specialist physician | Referral, counter-referral | No | No | Common chronic NCD | Health education, linkage, monitoring, medication management |

| **Appendix F: Studies identified at specialty outpatient clinics** | | | | | | | | | | | |
| --- | --- | --- | --- | --- | --- | --- | --- | --- | --- | --- | --- |
| **Key** | **Urban/ rural** | **Scale** | **Institution** | **Research role** | **Integration level** | **Primary provider** | **Linkage** | **Task shifting** | **Decent- ralization** | **Condition category** | **Services** |
| **Low HIV prevalence** | | | | | | | | | | | |
| *Lower middle-income countries* | | | | | | | | | | | |
| Ali India A[156] | Urban | Small to medium scale | Private | Experimental study | New care delivery teams | Multidisciplinary team | No | No | No | Common chronic NCD, common NP | Health education, screening, adherence support, psychotherapy, medication dispensing, patient follow-up, monitoring, medication management |
| Ali India Pakistan[182] | Urban | Small to medium scale | Public, private | Experimental study | New services integrated into existing delivery models | Midlevel provider | No | No | No | Common chronic NCD | Health education, adherence support, patient follow-up |
| *Upper middle-income countries* | | | | | | | | | | | |
| Luciano Brazil[183] | Urban | Single center | Public | Imbedded or evaluation | Description of existing delivery model | Multidisciplinary team | No | No | No | Common chronic NCD | Medication dispensing, monitoring |
| Jardim Brazil[184] | Not specified | Single center | Public | Imbedded or evaluation | Description of existing delivery model | Multispecialty, multi-cadre team | referral | No | No | Common chronic NCD | Health education, linkage, medication dispensing, patient follow-up, monitoring, medication management |
| Ali Colombia[185] | Urban | Single center | Public | Imbedded or evaluation | New care delivery teams | Specialist physician | No | No | No | Common chronic NCD | Health education, adherence support, medication dispensing, patient follow-up, medication management |
| Sharifi Iran B[90] | Urban | Small to medium scale | Public, private | Experimental study | New services integrated into existing delivery models | Specialist physician | Counter-referral | Yes | Yes | Common NP, severe NP | Linkage, patient follow-up, medication management , medication management |
| Chan Malaysia[186] | Not specified | Single center | Public | Experimental study | New services integrated into existing delivery models | Multidisciplinary team | No | No | No | Severe chronic NCD | Health education, adherence support |
| **High HIV prevalence** | | | | | | | | | | | |
| *Low-income countries* | | | | | | | | | | | |
| Bukirwa Uganda[187] | Not specified | Single center | NGO | Imbedded or evaluation | New conditions integrated into existing delivery models | Midlevel provider | Referral | No | No | Severe chronic NCD, chronic infection | Health education, screening, linkage |
| Nakimuli-Mpungu Uganda[188] | Urban | Single center | Not specified | Pilot | New conditions and new services integrated into existing delivery models | Not specified | Not specified | No | No | Common NP, chronic infection | Psychotherapy, patient follow-up |
| *Upper middle-income countries* | | | | | | | | | | | |
| Derman South Africa[189] | Urban | Single center | Not specified | Pilot | New conditions and new services integrated into existing delivery models | Multidisciplinary team | No | No | No | Common chronic NCD, severe chronic NCD | Health education, screening, peer-group facilitation, monitoring |

References to included studies

1. Rawal L, Jubayer S, Choudhury SR, Islam SMS, Abdullah AS: **Community health workers for non-communicable diseases prevention and control in Bangladesh: a qualitative study**. *Glob Health Res Policy* 2020, **6**(1):1.

2. Nimgaonkar V, Krishnamurti L, Prabhakar H, Menon N: **Comprehensive integrated care for patients with sickle cell disease in a remote aboriginal tribal population in southern India**. *Pediatr Blood Cancer* 2014, **61**(4):702-705.

3. Chatterjee S, Leese M, Koschorke M, McCrone P, Naik S, John S, Dabholkar H, Goldsmith K, Balaji M, Varghese M *et al*: **Collaborative community based care for people and their families living with schizophrenia in India: protocol for a randomised controlled trial**. *Trials* 2011, **12**:12.

4. Joshi R, Agrawal T, Fathima F, Usha T, Thomas T, Misquith D, Kalantri S, Chidambaram N, Raj T, Singamani A *et al*: **Cardiovascular risk factor reduction by community health workers in rural India: A cluster randomized trial**. *Am Heart J* 2019, **216**:9-19.

5. Amarchand R, Krishnan A, Saraf DS, Mathur P, Shukla DK, Nath LM: **Lessons for addressing noncommunicable diseases within a primary health-care system from the Ballabgarh project, India**. *WHO South East Asia J Public Health* 2015, **4**(2):130-138.

6. Basu P, Mahajan M, Patira N, Prasad S, Mogri S, Muwonge R, Lucas E, Sankaranarayanan R, Iyer S, Naik N *et al*: **A pilot study to evaluate home-based screening for the common non-communicable diseases by a dedicated cadre of community health workers in a rural setting in India**. *BMC Public Health* 2019, **19**(1):14.

7. Abdel-All M, Abimbola S, Praveen D, Joshi R: **What do Accredited Social Health Activists need to provide comprehensive care that incorporates non-communicable diseases? Findings from a qualitative study in Andhra Pradesh, India**. *Hum Resour Health* 2019, **17**(1):73.

8. Sahu B, Tn S, Hazra A: **Sustainability of barefoot nurse (BFN) project - Screening NCD and ensuring livelihood: A randomized control trial**. *Contemp Clin Trials Commun* 2020, **19**:100602.

9. Rawal LB, Kharel C, Yadav UN, Kanda K, Biswas T, Vandelanotte C, Baral S, Abdullah AS: **Community health workers for non-communicable disease prevention and control in Nepal: a qualitative study**. *BMJ Open* 2020, **10**(12):e040350.

10. Gyawali B, Neupane D, Vaidya A, Sandbæk A, Kallestrup P: **Community-based intervention for management of diabetes in Nepal (COBIN-D trial): study protocol for a cluster-randomized controlled trial**. *Trials* 2018, **19**(1):579.

11. Neupane G, Acharya S, Bhattarai M, Upadhyay A, Belbase B, Bhandari M, Pandeya D, Pokharel S, Ghimire S, Thapa G *et al*: **Study, Design, and Rationale of Noncommunicable Diseases in Nepal (NCD Nepal) Study: A Community-Based Prospective Epidemiological and Implementation Study in Rural Nepal**. *Glob Adv Health Med* 2020, **9**:2164956120917379.

12. Welton-Mitchell C, James LE, Khanal SN, James AS: **An integrated approach to mental health and disaster preparedness: a cluster comparison with earthquake affected communities in Nepal**. *BMC Psychiatry* 2018, **18**(1):296.

13. Ku GM, Kegels G: **Integrating chronic care with primary care activities: enriching healthcare staff knowledge and skills and improving glycemic control of a cohort of people with diabetes through the First Line Diabetes Care Project in the Philippines**. *Glob Health Action* 2014, **7**:25286.

14. Fernando N, Suveendran T, de Silva C: **Decentralizing provision of mental health care in Sri Lanka**. *WHO South East Asia J Public Health* 2017, **6**(1):18-21.

15. Murphy J, Goldsmith CH, Jones W, Oanh PT, Nguyen VC: **The effectiveness of a Supported Self-management task-shifting intervention for adult depression in Vietnam communities: study protocol for a randomized controlled trial**. *Trials* 2017, **18**(1):209.

16. Nguyen QN, Pham ST, Nguyen VL, Weinehall L, Wall S, Bonita R, Byass P: **Effectiveness of community-based comprehensive healthy lifestyle promotion on cardiovascular disease risk factors in a rural Vietnamese population: a quasi-experimental study**. *BMC Cardiovasc Disord* 2012, **12**:56.

17. Van Minh H, Do YK, Bautista MA, Tuan Anh T: **Describing the primary care system capacity for the prevention and management of non-communicable diseases in rural Vietnam**. *Int J Health Plann Manage* 2014, **29**(2):e159-173.

18. Falcone VM, Mäder CV, Nascimento CF, Santos JM, de Nóbrega FJ: **[Multiprofessional care and mental health in pregnant women]**. *Rev Saude Publica* 2005, **39**(4):612-618.

19. Struckmann V, Barbabella F, Dimova A, van Ginneken E: **Integrated Diabetes Care Delivered by Patients - A Case Study from Bulgaria**. *Int J Integr Care* 2017, **17**(1):6.

20. Chen Y, Yau E, Lam C, Deng H, Weng Y, Liu T, Mo X: **A 6-Month Randomized Controlled Pilot Study on the Effects of the Clubhouse Model of Psychosocial Rehabilitation with Chinese Individuals with Schizophrenia**. *Adm Policy Ment Health* 2020, **47**(1):107-114.

21. Zhong X, Wang Z, Fisher EB, Tanasugarn C: **Peer Support for Diabetes Management in Primary Care and Community Settings in Anhui Province, China**. *Ann Fam Med* 2015, **13 Suppl 1**:S50-58.

22. Kim YM, Ati A, Kols A, Lambe FM, Soetikno D, Wysong M, Tergas AI, Rajbhandari P, Lu E: **Influencing women's actions on cervical cancer screening and treatment in Karawang District, Indonesia**. *Asian Pac J Cancer Prev* 2012, **13**(6):2913-2921.

23. Tapia-Conyer R, Saucedo-Martínez R, Mújica-Rosales R, Gallardo-Rincón H, Lee E, Waugh C, Guajardo L, Torres-Beltrán B, Quijano-González Ú, López-Mendez M *et al*: **A Policy Analysis on the Proactive Prevention of Chronic Disease: Learnings from the Initial Implementation of Integrated Measurement for Early Detection (MIDO)**. *Int J Health Policy Manag* 2017, **6**(6):339-344.

24. Worster DT, Franke MF, Bazúa R, Flores H, García Z, Krupp J, Maza J, Palazuelos L, Rodríguez K, Newman PM *et al*: **Observational stepped-wedge analysis of a community health worker-led intervention for diabetes and hypertension in rural Mexico**. *BMJ Open* 2020, **10**(3):e034749.

25. Jiamjariyapon T, Ingsathit A, Pongpirul K, Vipattawat K, Kanchanakorn S, Saetie A, Kanistanon D, Wongprompitak P, Leesmidt V, Watcharasaksilp W *et al*: **Effectiveness of Integrated Care on Delaying Progression of stage 3-4 Chronic Kidney Disease in Rural Communities of Thailand (ESCORT study): a cluster randomized controlled trial**. *BMC Nephrol* 2017, **18**(1):83.

26. Turnacilar M, Sancar M, Apikoglu-Rabus S, Hursitoglu M, Izzettin FV: **Improvement of diabetes indices of care by a short pharmaceutical care program**. *Pharm World Sci* 2009, **31**(6):689-695.

27. Shields-Zeeman L, Petrea I, Smit F, Walters BH, Dedovic J, Kuzman MR, Nakov V, Nica R, Novotni A, Roth C *et al*: **Towards community-based and recovery-oriented care for severe mental disorders in Southern and Eastern Europe: aims and design of a multi-country implementation and evaluation study (RECOVER-E)**. *Int J Ment Health Syst* 2020, **14**:30.

28. Fekadu A, Hanlon C, Medhin G, Alem A, Selamu M, Giorgis TW, Shibre T, Teferra S, Tegegn T, Breuer E *et al*: **Development of a scalable mental healthcare plan for a rural district in Ethiopia**. *Br J Psychiatry* 2016, **208 Suppl 56**(Suppl 56):s4-12.

29. Smith SL, Misago CN, Osrow RA, Franke MF, Iyamuremye JD, Dusabeyezu JD, Mohand AA, Anatole M, Kayiteshonga Y, Raviola GJ: **Evaluating process and clinical outcomes of a primary care mental health integration project in rural Rwanda: a prospective mixed-methods protocol**. *BMJ Open* 2017, **7**(2):e014067.

30. Ngaruiya C, Oti S, van de Vijver S, Kyobutungi C, Free C: **Target women: Equity in access to mHealth technology in a non-communicable disease care intervention in Kenya**. *PLoS One* 2019, **14**(9):e0220834.

31. Pastakia SD, Ali SM, Kamano JH, Akwanalo CO, Ndege SK, Buckwalter VL, Vedanthan R, Bloomfield GS: **Screening for diabetes and hypertension in a rural low income setting in western Kenya utilizing home-based and community-based strategies**. *Global Health* 2013, **9**:21.

32. Rachlis B, Naanyu V, Wachira J, Genberg B, Koech B, Kamene R, Akinyi J, Braitstein P: **Identifying common barriers and facilitators to linkage and retention in chronic disease care in western Kenya**. *BMC Public Health* 2016, **16**:741.

33. Rachlis B, Naanyu V, Wachira J, Genberg B, Koech B, Kamene R, Akinyi J, Braitstein P: **Community Perceptions of Community Health Workers (CHWs) and Their Roles in Management for HIV, Tuberculosis and Hypertension in Western Kenya**. *PLoS One* 2016, **11**(2):e0149412.

34. Angwenyi V, Bunders-Aelen J, Criel B, Lazarus JV, Aantjes C: **An evaluation of self-management outcomes among chronic care patients in community home-based care programmes in rural Malawi: A 12-month follow-up study**. *Health Soc Care Community* 2020.

35. Kauye F, Chiwandira C, Wright J, Common S, Phiri M, Mafuta C, Maliwichi-Senganimalunje L, Udedi M: **Increasing the capacity of health surveillance assistants in community mental health care in a developing country, Malawi**. *Malawi Med J* 2011, **23**(3):85-88.

36. Kachimanga C, Cundale K, Wroe E, Nazimera L, Jumbe A, Dunbar E, Kalanga N: **Novel approaches to screening for noncommunicable diseases: Lessons from Neno, Malawi**. *Malawi Med J* 2017, **29**(2):78-83.

37. Banda HT, Mortimer K, Bello GA, Mbera GB, Namakhoma I, Thomson R, Nyirenda MJ, Faragher B, Madan J, Malmborg R *et al*: **Informal Health Provider and Practical Approach to Lung Health interventions to improve the detection of chronic airways disease and tuberculosis at primary care level in Malawi: study protocol for a randomised controlled trial**. *Trials* 2015, **16**:576.

38. Kwarisiima D, Atukunda M, Owaraganise A, Chamie G, Clark T, Kabami J, Jain V, Byonanebye D, Mwangwa F, Balzer LB *et al*: **Hypertension control in integrated HIV and chronic disease clinics in Uganda in the SEARCH study**. *BMC Public Health* 2019, **19**(1):511.

39. Chamie G, Kwarisiima D, Clark TD, Kabami J, Jain V, Geng E, Petersen ML, Thirumurthy H, Kamya MR, Havlir DV *et al*: **Leveraging rapid community-based HIV testing campaigns for non-communicable diseases in rural Uganda**. *PLoS One* 2012, **7**(8):e43400.

40. Jack BA, Kirton J, Birakurataki J, Merriman A: **'A bridge to the hospice': the impact of a Community Volunteer Programme in Uganda**. *Palliat Med* 2011, **25**(7):706-715.

41. Dunbar EL, Wroe EB, Nhlema B, Kachimanga C, Gupta R, Taylor C, Michaelis A, Cundale K, Dullie L, Jumbe A *et al*: **Evaluating the impact of a community health worker programme on non-communicable disease, malnutrition, tuberculosis, family planning and antenatal care in Neno, Malawi: protocol for a stepped-wedge, cluster randomised controlled trial**. *BMJ Open* 2018, **8**(7):e019473.

42. Hartwig KN, Hartwig KA, DiSorbo P, Hofgren B, Motz-Storey L, Mmbando P, Msurri M, Mwangi-Powell F, Powell RA, Smith S *et al*: **Scaling up a community-based palliative care program among faith-based hospitals in Tanzania**. *J Palliat Care* 2010, **26**(3):194-201.

43. Duffy M, Sharer M, Cornman H, Pearson J, Pitorak H, Fullem A: **Integrating Mental Health and HIV Services in Zimbabwean Communities: A Nurse and Community-led Approach to Reach the Most Vulnerable**. *J Assoc Nurses AIDS Care* 2017, **28**(2):186-198.

44. Petersen I, Bhana A, Campbell-Hall V, Mjadu S, Lund C, Kleintjies S, Hosegood V, Flisher AJ: **Planning for district mental health services in South Africa: a situational analysis of a rural district site**. *Health Policy Plan* 2009, **24**(2):140-150.

45. Madela S, James S, Sewpaul R, Madela S, Reddy P: **Early detection, care and control of hypertension and diabetes in South Africa: A community-based approach**. *Afr J Prim Health Care Fam Med* 2020, **12**(1):e1-e9.

46. Schnippel K, Lince-Deroche N, van den Handel T, Molefi S, Bruce S, Firnhaber C: **Cost evaluation of reproductive and primary health care mobile service delivery for women in two rural districts in South Africa**. *PLoS One* 2015, **10**(3):e0119236.

47. O'Brien V, Jenkins LS, Munnings M, Grey H, North Z, Schumann H, De Klerk-Green E: **Palliative care made visible: Developing a rural model for the Western Cape Province, South Africa**. *Afr J Prim Health Care Fam Med* 2019, **11**(1):e1-e11.

48. Ndou T, van Zyl G, Hlahane S, Goudge J: **A rapid assessment of a community health worker pilot programme to improve the management of hypertension and diabetes in Emfuleni sub-district of Gauteng Province, South Africa**. *Glob Health Action* 2013, **6**:19228.

49. Govindasamy D, Kranzer K, van Schaik N, Noubary F, Wood R, Walensky RP, Freedberg KA, Bassett IV, Bekker LG: **Linkage to HIV, TB and non-communicable disease care from a mobile testing unit in Cape Town, South Africa**. *PLoS One* 2013, **8**(11):e80017.

50. Müller I, Smith D, Adams L, Aerts A, Damons BP, Degen J, Gall S, Gani Z, Gerber M, Gresse A *et al*: **Effects of a School-Based Health Intervention Program in Marginalized Communities of Port Elizabeth, South Africa (the KaziBantu Study): Protocol for a Randomized Controlled Trial**. *JMIR Res Protoc* 2019, **8**(7):e14097.

51. Golovaty I, Sharma M, Van Heerden A, van Rooyen H, Baeten JM, Celum C, Barnabas RV: **Cost of Integrating Noncommunicable Disease Screening Into Home-Based HIV Testing and Counseling in South Africa**. *J Acquir Immune Defic Syndr* 2018, **78**(5):522-526.

52. Morris-Paxton AA, Rheeder P, Ewing RG, Woods D: **Detection, referral and control of diabetes and hypertension in the rural Eastern Cape Province of South Africa by community health outreach workers in the rural primary healthcare project: Health in Every Hut**. *Afr J Prim Health Care Fam Med* 2018, **10**(1):e1-e8.

53. Botha UA, Koen L, Joska JA, Hering LM, Oosthuizen PP: **Assessing the efficacy of a modified assertive community-based treatment programme in a developing country**. *BMC Psychiatry* 2010, **10**:73.

54. Siedner MJ, Baisley K, Orne-Gliemann J, Pillay D, Koole O, Wong EB, Matthews P, Tanser F, Herbst K, Barnighausen T *et al*: **Linkage to primary care after home-based blood pressure screening in rural KwaZulu-Natal, South Africa: a population-based cohort study**. *BMJ Open* 2018, **8**(12):e023369.

55. Kutcher S, Perkins K, Gilberds H, Udedi M, Ubuguyu O, Njau T, Chapota R, Hashish M: **Creating Evidence-Based Youth Mental Health Policy in Sub-Saharan Africa: A Description of the Integrated Approach to Addressing the Issue of Youth Depression in Malawi and Tanzania**. *Front Psychiatry* 2019, **10**:542.

56. Guwatudde D, Absetz P, Delobelle P, Östenson CG, Olmen Van J, Alvesson HM, Mayega RW, Ekirapa Kiracho E, Kiguli J, Sundberg CJ *et al*: **Study protocol for the SMART2D adaptive implementation trial: a cluster randomised trial comparing facility-only care with integrated facility and community care to improve type 2 diabetes outcomes in Uganda, South Africa and Sweden**. *BMJ Open* 2018, **8**(3):e019981.

57. Dorji T, Yangchen P, Dorji C, Nidup T, Zam K: **An approach to diabetes prevention and management: The Bhutan experience**. *WHO South East Asia J Public Health* 2016, **5**(1):44-47.

58. Khan MA, Walley JD, Khan N, Khan MA, Ali S, King R, Khan SE, Sheikh FI, Manzoor F, Khan HJ: **Delivering integrated hypertension care at private health facilities in urban Pakistan: a process evaluation**. *BJGP Open* 2018, **2**(4):bjgpopen18X101613.

59. Khan MA, Khan N, Walley JD, Khan MA, Hicks J, Ahmed M, Sheikh FI, Ali M, Manzoor F, Khan HJ: **Effectiveness of delivering integrated COPD care at public healthcare facilities: a cluster randomised trial in Pakistan**. *BJGP Open* 2019, **3**(1):bjgpopen18X101634.

60. Khan MA, Walley JD, Khan N, Hicks J, Ahmed M, Khan SE, Khan MA, Khan HJ, Harries AD: **Effectiveness of an integrated diabetes care package at primary healthcare facilities: a cluster randomised trial in Pakistan**. *BJGP Open* 2018, **2**(4):bjgpopen18X101618.

61. Lall D, Engel N, Devadasan N, Horstman K, Criel B: **Challenges in primary care for diabetes and hypertension: an observational study of the Kolar district in rural India**. *BMC Health Serv Res* 2019, **19**(1):44.

62. Lall D, Engel N, Devadasan N, Horstman K, Criel B: **Team-based primary health care for non-communicable diseases: complexities in South India**. *Health Policy Plan* 2020, **35**(Supplement_2):ii22-ii34.

63. Mallawaarachchi DSV, Wickremasinghe SC, Somatunga LC, Siriwardena VT, Gunawardena NS: **Healthy Lifestyle Centres: a service for screening noncommunicable diseases through primary health-care institutions in Sri Lanka**. *WHO South East Asia J Public Health* 2016, **5**(2):89-95.

64. Manjunatha N, Singh G: **Manochaitanya: integrating mental health into primary health care**. *Lancet* 2016, **387**(10019):647-648.

65. Murphy J, Corbett KK, Linh DT, Oanh PT, Nguyen VC: **Barriers and facilitators to the integration of depression services in primary care in Vietnam: a mixed methods study**. *BMC Health Serv Res* 2018, **18**(1):641.

66. Piette JD, Valverde H, Marinec N, Jantz R, Kamis K, de la Vega CL, Woolley T, Pinto B: **Establishing an independent mobile health program for chronic disease self-management support in bolivia**. *Front Public Health* 2014, **2**:95.

67. Spagnolo J, Champagne F, Leduc N, Piat M, Melki W, Charfi F, Laporta M: **Building system capacity for the integration of mental health at the level of primary care in Tunisia: a study protocol in global mental health**. *BMC Health Serv Res* 2017, **17**(1):38.

68. Wangchuk D, Virdi NK, Garg R, Mendis S, Nair N, Wangchuk D, Kumar R: **Package of essential noncommunicable disease (PEN) interventions in primary health-care settings of Bhutan: a performance assessment study**. *WHO South East Asia J Public Health* 2014, **3**(2):154-160.

69. Anjara SG, Bonetto C, Ganguli P, Setiyawati D, Mahendradhata Y, Yoga BH, Trisnantoro L, Brayne C, Van Bortel T: **Can General Practitioners manage mental disorders in primary care? A partially randomised, pragmatic, cluster trial**. *PLoS One* 2019, **14**(11):e0224724.

70. Arevian M: **The significance of a collaborative practice model in delivering care to chronically ill patients: a case study of managing diabetes mellitus in a primary health care center**. *J Interprof Care* 2005, **19**(5):444-451.

71. Barceló A, Cafiero E, de Boer M, Mesa AE, Lopez MG, Jiménez RA, Esqueda AL, Martinez JA, Holguin EM, Meiners M *et al*: **Using collaborative learning to improve diabetes care and outcomes: the VIDA project**. *Prim Care Diabetes* 2010, **4**(3):145-153.

72. Beganlic A, Pavljasevic S, Kreitmayer S, Zildzic M, Softic A, Selmanovic S, Becarevic M: **Qualitative Evaluation of Cardiovascular Diseases Management in Family Medicine Team in One Year Level**. *Med Arch* 2015, **69**(3):140-144.

73. Cerci Neto A, Ferreira Filho OF, Bueno T, Talhari MA: **Reduction in the number of asthma-related hospital admissions after the implementation of a multidisciplinary asthma control program in the city of Londrina, Brazil**. *J Bras Pneumol* 2008, **34**(9):639-645.

74. Chen S, Conwell Y, Xu B, Chiu H, Tu X, Ma Y: **Depression care management for late-life depression in China primary care: protocol for a randomized controlled trial**. *Trials* 2011, **12**:121.

75. Chen S, Burström B, Sparring V, Qian D: **Vertical integrated service model: an educational intervention for chronic disease management and its effects in rural China - a study protocol**. *BMC Health Serv Res* 2018, **18**(1):567.

76. Chua SS, Kok LC, Yusof FA, Tang GH, Lee SW, Efendie B, Paraidathathu T: **Pharmaceutical care issues identified by pharmacists in patients with diabetes, hypertension or hyperlipidaemia in primary care settings**. *BMC Health Serv Res* 2012, **12**:388.

77. Cueto-Manzano AM, Martínez-Ramírez HR, Cortés-Sanabria L: **Management of chronic kidney disease: primary health-care setting, self-care and multidisciplinary approach**. *Clin Nephrol* 2010, **74 Suppl 1**:S99-104.

78. da Silva Marinho MG, Fontbonne A, Vasconcelos Barbosa JM, de Melo Rodrigues H, Freese de Carvalho E, Vieira de Souza W, Pessoa Cesse EA: **The impact of an intervention to improve diabetes management in primary healthcare professionals' practices in Brazil**. *Prim Care Diabetes* 2017, **11**(6):538-545.

79. Didier MT, Guimarães AC: **Optimizing the treatment of hypertension in the primary care setting**. *Arq Bras Cardiol* 2007, **88**(2):218-224.

80. Gagliardino JJ, Olivera E, Etchegoyen GS, Guidi ML, Caporale JE, Martella A, Hera Mde L, Siri F, Bonelli P: **PROPAT: a study to improve the quality and reduce the cost of diabetes care**. *Diabetes Res Clin Pract* 2006, **72**(3):284-291.

81. Jiao F, Fung CS, Wan YF, McGhee SM, Wong CK, Dai D, Kwok R, Lam CL: **Long-term effects of the multidisciplinary risk assessment and management program for patients with diabetes mellitus (RAMP-DM): a population-based cohort study**. *Cardiovasc Diabetol* 2015, **14**:105.

82. Kuhmmer R, Lazzaretti RK, Guterres CM, Raimundo FV, Leite LE, Delabary TS, Caon S, Bastos GA, Polanczyk CA: **Effectiveness of multidisciplinary intervention on blood pressure control in primary health care: a randomized clinical trial**. *BMC Health Serv Res* 2016, **16**(1):456.

83. Li LW, Xue J, Conwell Y, Yang Q, Chen S: **Implementing collaborative care for older people with comorbid hypertension and depression in rural China**. *Int Psychogeriatr* 2019:1-9.

84. Martins SM, Salibe-Filho W, Tonioli LP, Pfingesten LE, Braz PD, McDonnell J, Williams S, do Carmo D, de Sousa JC, Pinnock H *et al*: **Implementation of 'matrix support' (collaborative care) to reduce asthma and COPD referrals and improve primary care management in Brazil: a pilot observational study**. *NPJ Prim Care Respir Med* 2016, **26**:16047.

85. Miao Y, Zhang L, Sparring V, Sandeep S, Tang W, Sun X, Feng D, Ye T: **Improving health related quality of life among rural hypertensive patients through the integrative strategy of health services delivery: a quasi-experimental trial from Chongqing, China**. *Int J Equity Health* 2016, **15**(1):132.

86. Mino-León D, Reyes-Morales H, Flores-Hernández S: **Effectiveness of involving pharmacists in the process of ambulatory health care to improve drug treatment adherence and disease control**. *J Eval Clin Pract* 2015, **21**(1):7-12.

87. Pilipovic-Broceta N, Vasiljevic N, Marinkovic J, Todorovic N, Jankovic J, Ostric I, Kalimanovska-Ostric D, Racic M: **Assessment of hypertension chronic care model: Pacic application in Bosnia and Herzegovina**. *PLoS One* 2018, **13**(8):e0202250.

88. Prestes M, Gayarre MA, Elgart JF, Gonzalez L, Rucci E, Gagliardino JJ: **Multistrategic approach to improve quality of care of people with diabetes at the primary care level: Study design and baseline data**. *Prim Care Diabetes* 2017, **11**(2):193-200.

89. Ramli AS, Selvarajah S, Daud MH, Haniff J, Abdul-Razak S, Tg-Abu-Bakar-Sidik TM, Bujang MA, Chew BH, Rahman T, Tong SF *et al*: **Effectiveness of the EMPOWER-PAR Intervention in Improving Clinical Outcomes of Type 2 Diabetes Mellitus in Primary Care: A Pragmatic Cluster Randomised Controlled Trial**. *BMC Fam Pract* 2016, **17**(1):157.

90. Sharifi V, Shahrivar Z, Zarafshan H, Ashkezary SB, Stuart E, Mojtabai R, Wissow L: **Collaborative care for child and youth mental health problems in a middle-income country: study protocol for a randomized controlled trial training general practitioners**. *Trials* 2019, **20**(1):405.

91. Shi L, Makinen M, Lee DC, Kidane R, Blanchet N, Liang H, Li J, Lindelow M, Wang H, Xie S *et al*: **Integrated care delivery and health care seeking by chronically-ill patients - a case-control study of rural Henan province, China**. *Int J Equity Health* 2015, **14**:98.

92. Torrey WC, Cepeda M, Castro S, Bartels SM, Cubillos L, Obando FS, Camblor PM, Uribe-Restrepo JM, Williams M, Gómez-Restrepo C *et al*: **Implementing Technology-Supported Care for Depression and Alcohol Use Disorder in Primary Care in Colombia: Preliminary Findings**. *Psychiatr Serv* 2020, **71**(7):678-683.

93. Tu Q, Xiao LD, Ullah S, Fuller J, Du H: **A transitional care intervention for hypertension control for older people with diabetes: A cluster randomized controlled trial**. *J Adv Nurs* 2020.

94. Wesseling C, Román N, Quirós I, Páez L, García V, Mora AM, Juncos JL, Steenland KN: **Parkinson's and Alzheimer's diseases in Costa Rica: a feasibility study toward a national screening program**. *Glob Health Action* 2013, **6**:23061.

95. Wong CK, Wong WC, Lam CL, Wan YF, Wong WH, Chung KL, Dai D, Tsui EL, Fong DY: **Effects of Patient Empowerment Programme (PEP) on clinical outcomes and health service utilization in type 2 diabetes mellitus in primary care: an observational matched cohort study**. *PLoS One* 2014, **9**(5):e95328.

96. Xu L, Fang WY, Zhu F, Zhang HG, Liu K: **A coordinated PCP-Cardiologist Telemedicine Model (PCTM) in China's community hypertension care: study protocol for a randomized controlled trial**. *Trials* 2017, **18**(1):236.

97. Yin J, Wei X, Li H, Jiang Y, Mao C: **Assessing the impact of general practitioner team service on perceived quality of care among patients with non-communicable diseases in China: a natural experimental study**. *Int J Qual Health Care* 2016, **28**(5):554-560.

98. Zhu X, Wong FKY, Wu CLH: **Development and evaluation of a nurse-led hypertension management model: A randomized controlled trial**. *Int J Nurs Stud* 2018, **77**:171-178.

99. Labhardt ND, Balo JR, Ndam M, Grimm JJ, Manga E: **Task shifting to non-physician clinicians for integrated management of hypertension and diabetes in rural Cameroon: a programme assessment at two years**. *BMC Health Serv Res* 2010, **10**:339.

100. Mamo Y, Seid E, Adams S, Gardiner A, Parry E: **A primary healthcare approach to the management of chronic disease in Ethiopia: an example for other countries**. *Clin Med (Lond)* 2007, **7**(3):228-231.

101. Ndayisaba A, Harerimana E, Borg R, Miller AC, Kirk CM, Hann K, Hirschhorn LR, Manzi A, Ngoga G, Dusabeyezu S *et al*: **A Clinical Mentorship and Quality Improvement Program to Support Health Center Nurses Manage Type 2 Diabetes in Rural Rwanda**. *J Diabetes Res* 2017, **2017**:2657820.

102. Zou G, Witter S, Caperon L, Walley J, Cheedella K, Senesi RGB, Wurie HR: **Adapting and implementing training, guidelines and treatment cards to improve primary care-based hypertension and diabetes management in a fragile context: results of a feasibility study in Sierra Leone**. *BMC Public Health* 2020, **20**(1):1185.

103. Adewuya AO, Adewumi T, Momodu O, Olibamoyo O, Adesoji O, Adegbokun A, Adeyemo S, Manuwa O, Adegbaju D: **Development and feasibility assessment of a collaborative stepped care intervention for management of depression in the mental health in primary care (MeHPriC) project, Lagos, Nigeria**. *Psychol Med* 2019, **49**(13):2149-2157.

104. Adler AJ, Laar A, Prieto-Merino D, Der RMM, Mangortey D, Dirks R, Lamptey P, Perel P: **Can a nurse-led community-based model of hypertension care improve hypertension control in Ghana? Results from the ComHIP cohort study**. *BMJ Open* 2019, **9**(4):e026799.

105. Claeys P, De Vuyst H, Mzenge G, Sande J, Dhondt V, Temmerman M: **Integration of cervical screening in family planning clinics**. *Int J Gynaecol Obstet* 2003, **81**(1):103-108.

106. Gureje O, Makanjuola V, Kola L, Yusuf B, Price L, Esan O, Oladeji BD, Appiah-Poku J, Haris B, Othieno C *et al*: **COllaborative Shared care to IMprove Psychosis Outcome (COSIMPO): study protocol for a randomized controlled trial**. *Trials* 2017, **18**(1):462.

107. Gureje O, Abdulmalik J, Kola L, Musa E, Yasamy MT, Adebayo K: **Integrating mental health into primary care in Nigeria: report of a demonstration project using the mental health gap action programme intervention guide**. *BMC Health Serv Res* 2015, **15**:242.

108. Huchko MJ, Bukusi EA, Cohen CR: **Building capacity for cervical cancer screening in outpatient HIV clinics in the Nyanza province of western Kenya**. *Int J Gynaecol Obstet* 2011, **114**(2):106-110.

109. Jenkins R, Kiima D, Njenga F, Okonji M, Kingora J, Kathuku D, Lock S: **Integration of mental health into primary care in Kenya**. *World Psychiatry* 2010, **9**(2):118-120.

110. Kengne AP, Sobngwi E, Fezeu L, Awah PK, Dongmo S, Mbanya JC: **Setting-up nurse-led pilot clinics for the management of non-communicable diseases at primary health care level in resource-limited settings of Africa**. *Pan Afr Med J* 2009, **3**:10.

111. Khabala KB, Edwards JK, Baruani B, Sirengo M, Musembi P, Kosgei RJ, Walter K, Kibachio JM, Tondoi M, Ritter H *et al*: **Medication Adherence Clubs: a potential solution to managing large numbers of stable patients with multiple chronic diseases in informal settlements**. *Trop Med Int Health* 2015, **20**(10):1265-1270.

112. Kumar M, Huang KY, Othieno C, Wamalwa D, Hoagwood K, Unutzer J, Saxena S, Petersen I, Njuguna S, Amugune B *et al*: **Implementing combined WHO mhGAP and adapted group interpersonal psychotherapy to address depression and mental health needs of pregnant adolescents in Kenyan primary health care settings (INSPIRE): a study protocol for pilot feasibility trial of the integrated intervention in LMIC settings**. *Pilot Feasibility Stud* 2020, **6**:136.

113. Nnodu OE, Sopekan A, Nnebe-Agumadu U, Ohiaeri C, Adeniran A, Shedul G, Isa HA, Owolabi O, Chianumba RI, Tanko Y *et al*: **Implementing newborn screening for sickle cell disease as part of immunisation programmes in Nigeria: a feasibility study**. *Lancet Haematol* 2020, **7**(7):e534-e540.

114. Osetinsky B, Genberg BL, Bloomfield GS, Hogan J, Pastakia S, Sang E, Ngressa A, Mwangi A, Lurie MN, McGarvey ST *et al*: **Hypertension Control and Retention in Care Among HIV-Infected Patients: The Effects of Co-located HIV and Chronic Noncommunicable Disease Care**. *J Acquir Immune Defic Syndr* 2019, **82**(4):399-406.

115. Some D, Edwards JK, Reid T, Van den Bergh R, Kosgei RJ, Wilkinson E, Baruani B, Kizito W, Khabala K, Shah S *et al*: **Task Shifting the Management of Non-Communicable Diseases to Nurses in Kibera, Kenya: Does It Work?** *PLoS One* 2016, **11**(1):e0145634.

116. Gutnik L, Lee C, Msosa V, Moses A, Stanley C, Mzumara S, Liomba NG, Gopal S: **Clinical breast examination screening by trained laywomen in Malawi integrated with other health services**. *J Surg Res* 2016, **204**(1):61-67.

117. Muddu M, Tusubira AK, Nakirya B, Nalwoga R, Semitala FC, Akiteng AR, Schwartz JI, Ssinabulya I: **Exploring barriers and facilitators to integrated hypertension-HIV management in Ugandan HIV clinics using the Consolidated Framework for Implementation Research (CFIR)**. *Implement Sci Commun* 2020, **1**:45.

118. Wroe EB, Kalanga N, Mailosi B, Mwalwanda S, Kachimanga C, Nyangulu K, Dunbar E, Kerr L, Nazimera L, Dullie L: **Leveraging HIV platforms to work toward comprehensive primary care in rural Malawi: the Integrated Chronic Care Clinic**. *Healthc (Amst)* 2015, **3**(4):270-276.

119. Mwanahamuntu MH, Sahasrabuddhe VV, Pfaendler KS, Mudenda V, Hicks ML, Vermund SH, Stringer JS, Parham GP: **Implementation of 'see-and-treat' cervical cancer prevention services linked to HIV care in Zambia**. *AIDS* 2009, **23**(6):N1-5.

120. Chibanda D, Weiss HA, Verhey R, Simms V, Munjoma R, Rusakaniko S, Chingono A, Munetsi E, Bere T, Manda E *et al*: **Effect of a Primary Care-Based Psychological Intervention on Symptoms of Common Mental Disorders in Zimbabwe: A Randomized Clinical Trial**. *JAMA* 2016, **316**(24):2618-2626.

121. Frieden M, Zamba B, Mukumbi N, Mafaune PT, Makumbe B, Irungu E, Moneti V, Isaakidis P, Garone D, Prasai M: **Setting up a nurse-led model of care for management of hypertension and diabetes mellitus in a high HIV prevalence context in rural Zimbabwe: a descriptive study**. *BMC Health Serv Res* 2020, **20**(1):486.

122. Ameh S, Klipstein-Grobusch K, Musenge E, Kahn K, Tollman S, Gómez-Olivé FX: **Effectiveness of an Integrated Approach to HIV and Hypertension Care in Rural South Africa: Controlled Interrupted Time-Series Analysis**. *J Acquir Immune Defic Syndr* 2017, **75**(4):472-479.

123. Coleman R, Gill G, Wilkinson D: **Noncommunicable disease management in resource-poor settings: a primary care model from rural South Africa**. *Bull World Health Organ* 1998, **76**(6):633-640.

124. Fairall LR, Folb N, Timmerman V, Lombard C, Steyn K, Bachmann MO, Bateman ED, Lund C, Cornick R, Faris G *et al*: **Educational Outreach with an Integrated Clinical Tool for Nurse-Led Non-communicable Chronic Disease Management in Primary Care in South Africa: A Pragmatic Cluster Randomised Controlled Trial**. *PLoS Med* 2016, **13**(11):e1002178.

125. Hlongwa EN, Sibiya MN: **Challenges affecting the implementation of the Policy on Integration of Mental Health Care into primary healthcare in KwaZulu-Natal province**. *Curationis* 2019, **42**(1):e1-e9.

126. Khan T, Bertram MY, Jina R, Mash B, Levitt N, Hofman K: **Preventing diabetes blindness: cost effectiveness of a screening programme using digital non-mydriatic fundus photography for diabetic retinopathy in a primary health care setting in South Africa**. *Diabetes Res Clin Pract* 2013, **101**(2):170-176.

127. Lebina L, Alaba O, Kawonga M, Oni T: **Process evaluation of fidelity and costs of implementing the Integrated Chronic Disease Management model in South Africa: mixed methods study protocol**. *BMJ Open* 2019, **9**(6):e029277.

128. Lovero KL, Lammie SL, van Zyl A, Paul SN, Ngwepe P, Mootz JJ, Carlson C, Sweetland AC, Shelton RC, Wainberg ML *et al*: **Mixed-methods evaluation of mental healthcare integration into tuberculosis and maternal-child healthcare services of four South African districts**. *BMC Health Serv Res* 2019, **19**(1):83.

129. Lund C, Schneider M, Davies T, Nyatsanza M, Honikman S, Bhana A, Bass J, Bolton P, Dewey M, Joska J *et al*: **Task sharing of a psychological intervention for maternal depression in Khayelitsha, South Africa: study protocol for a randomized controlled trial**. *Trials* 2014, **15**:457.

130. Maconick L, Jenkins LS, Fisher H, Petrie A, Boon L, Reuter H: **Mental health in primary care: Integration through in-service training in a South African rural clinic**. *Afr J Prim Health Care Fam Med* 2018, **10**(1):e1-e7.

131. Mahomed OH, Asmall S: **Professional nurses' perceptions and experiences with the implementation of an integrated chronic care model at primary healthcare clinics in South Africa**. *Curationis* 2017, **40**(1):e1-e6.

132. Myers B, Lund C, Lombard C, Joska J, Levitt N, Butler C, Cleary S, Naledi T, Milligan P, Stein DJ *et al*: **Comparing dedicated and designated models of integrating mental health into chronic disease care: study protocol for a cluster randomized controlled trial**. *Trials* 2018, **19**(1):185.

133. Spedding M, Stein DJ, Naledi T, Myers B, Cuijpers P, Sorsdahl K: **A task-sharing intervention for prepartum common mental disorders: Feasibility, acceptability and responses in a South African sample**. *Afr J Prim Health Care Fam Med* 2020, **12**(1):e1-e9.

134. Ramogola-Masire D, de Klerk R, Monare B, Ratshaa B, Friedman HM, Zetola NM: **Cervical cancer prevention in HIV-infected women using the "see and treat" approach in Botswana**. *J Acquir Immune Defic Syndr* 2012, **59**(3):308-313.

135. Thorogood M, Goudge J, Bertram M, Chirwa T, Eldridge S, Gómez-Olivé FX, Limbani F, Musenge E, Myakayaka N, Tollman S *et al*: **The Nkateko health service trial to improve hypertension management in rural South Africa: study protocol for a randomised controlled trial**. *Trials* 2014, **15**:435.

136. Van Hout MC, Bachmann M, Lazarus JV, Shayo EH, Bukenya D, Picchio CA, Nyirenda M, Mfinanga SG, Birungi J, Okebe J *et al*: **Strengthening integration of chronic care in Africa: protocol for the qualitative process evaluation of integrated HIV, diabetes and hypertension care in a cluster randomised controlled trial in Tanzania and Uganda**. *BMJ Open* 2020, **10**(10):e039237.

137. Ansbro É M, Biringanine M, Caleo G, Prieto-Merino D, Sadique Z, Perel P, Jobanputra K, Roberts B: **Management of diabetes and associated costs in a complex humanitarian setting in the Democratic Republic of Congo: a retrospective cohort study**. *BMJ Open* 2019, **9**(11):e030176.

138. Huque R, Nasreen S, Ahmed F, Hicks JP, Walley J, Newell JN, Elsey H: **Integrating a diabetes and hypertension case management package within primary health care: a mixed methods feasibility study in Bangladesh**. *BMC Health Serv Res* 2018, **18**(1):811.

139. Shukla AK, Singh S, Sheikh A, Singh S, Gupta G, Daberao R: **Diabetic retinopathy screening at primary and community health centers in Maharashtra**. *Indian J Ophthalmol* 2020, **68**(Suppl 1):S83-s87.

140. Srinivasapura Venkateshmurthy N, Ajay VS, Mohan S, Jindal D, Anand S, Kondal D, Tandon N, Rao MB, Prabhakaran D: **m-Power Heart Project - a nurse care coordinator led, mHealth enabled intervention to improve the management of hypertension in India: study protocol for a cluster randomized trial**. *Trials* 2018, **19**(1):429.

141. Collins D, Inglin L, Laatikainen T, Ciobanu A, Curocichin G, Salaru V, Zatic T, Anisei A, Chiosa D, Munteanu M *et al*: **Implementing a package of noncommunicable disease interventions in the Republic of Moldova: two-year follow-up data**. *Prim Health Care Res Dev* 2020, **21**:e39.

142. Mourão CA, 2nd, de Sá JR, Guedes OM, Dib SA: **Glycemic control in adult type 1 diabetes patients from a brazilian country city: comparison between a multidisciplinary and a routine endocrinological approach**. *Arq Bras Endocrinol Metabol* 2006, **50**(5):944-950.

143. Li Y, Wang JL, Zhang XC, Liu D, Shi WH, Liang XF, Wu J: **Effectiveness of Adherence to Standardized Hypertension Management by Primary Health Care Workers in China: a Cross-sectional Survey 3 Years after the Healthcare Reform**. *Biomed Environ Sci* 2016, **29**(12):915-921.

144. Chao J, Yang L, Xu H, Yu Q, Jiang L, Zong M: **The effect of integrated health management model on the health of older adults with diabetes in a randomized controlled trial**. *Arch Gerontol Geriatr* 2015, **60**(1):82-88.

145. Chen S, Hou X, Sun Y, Hu G, Zhou X, Xue H, Chen P, Wu J, Bao Y, Jia W: **A seven-year study on an integrated hospital-community diabetes management program in Chinese patients with diabetes**. *Prim Care Diabetes* 2018, **12**(3):231-237.

146. Silva-Tinoco R, Cuatecontzi-Xochitiotzi T, De la Torre-Saldaña V, León-García E, Serna-Alvarado J, Guzmán-Olvera E, Cabrera D, Gay JG, Prada D: **Role of social and other determinants of health in the effect of a multicomponent integrated care strategy on type 2 diabetes mellitus**. *Int J Equity Health* 2020, **19**(1):75.

147. Walsh KF, Lee MH, Martelly S, Pierre MM, Joseph J, Gustin M, Peck RN, Fitzgerald DW, Pape JW, McNairy M: **Integrating hypertension services at an HIV clinic in Port-au-Prince, Haiti: A report from the field**. *J Clin Hypertens (Greenwich)* 2018, **20**(10):1485-1492.

148. Rusingiza EK, El-Khatib Z, Hedt-Gauthier B, Ngoga G, Dusabeyezu S, Tapela N, Mutumbira C, Mutabazi F, Harelimana E, Mucumbitsi J *et al*: **Outcomes for patients with rheumatic heart disease after cardiac surgery followed at rural district hospitals in Rwanda**. *Heart* 2018, **104**(20):1707-1713.

149. Eberly LA, Rusangwa C, Ng'ang'a L, Neal CC, Mukundiyukuri JP, Mpanusingo E, Mungunga JC, Habineza H, Anderson T, Ngoga G *et al*: **Cost of integrated chronic care for severe non-communicable diseases at district hospitals in rural Rwanda**. *BMJ Glob Health* 2019, **4**(3):e001449.

150. Eberly LA, Rusingiza E, Park PH, Ngoga G, Dusabeyezu S, Mutabazi F, Harerimana E, Mucumbitsi J, Nyembo PF, Borg R *et al*: **Nurse-Driven Echocardiography and Management of Heart Failure at District Hospitals in Rural Rwanda**. *Circ Cardiovasc Qual Outcomes* 2018, **11**(12):e004881.

151. Rutayisire R, Mutabazi F, Bayingana A, Miller AC, Gupta N, Ngoga G, Ngabireyimana E, Borg R, Rusingiza E, Bavuma C *et al*: **Integration of Chronic Oncology Services in Noncommunicable Disease Clinic in Rural Rwanda**. *Ann Glob Health* 2020, **86**(1):33.

152. Levy R, Mathai M, Chatterjee P, Ongeri L, Njuguna S, Onyango D, Akena D, Rota G, Otieno A, Neylan TC *et al*: **Implementation research for public sector mental health care scale-up (SMART-DAPPER): a sequential multiple, assignment randomized trial (SMART) of non-specialist-delivered psychotherapy and/or medication for major depressive disorder and posttraumatic stress disorder (DAPPER) integrated with outpatient care clinics at a county hospital in Kenya**. *BMC Psychiatry* 2019, **19**(1):424.

153. Odafe S, Torpey K, Khamofu H, Oladele E, Adedokun O, Chabikuli O, Mukaddas H, Usman Y, Aiyenigba B, Okoye M: **Integrating cervical cancer screening with HIV care in a district hospital in Abuja, Nigeria**. *Niger Med J* 2013, **54**(3):176-184.

154. Gaynes BN, Akiba CF, Hosseinipour MC, Kulisewa K, Amberbir A, Udedi M, Zimba CC, Masiye JK, Crampin M, Amarreh I *et al*: **The Sub-Saharan Africa Regional Partnership (SHARP) for Mental Health Capacity-Building Scale-Up Trial: Study Design and Protocol**. *Psychiatr Serv* 2020:appips202000003.

155. Talama GC, Shaw M, Maloya J, Chihana T, Nazimera L, Wroe EB, Kachimanga C: **Improving uptake of cervical cancer screening services for women living with HIV and attending chronic care services in rural Malawi**. *BMJ Open Qual* 2020, **9**(3).

156. Ali MK, Chwastiak L, Poongothai S, Emmert-Fees KMF, Patel SA, Anjana RM, Sagar R, Shankar R, Sridhar GR, Kosuri M *et al*: **Effect of a Collaborative Care Model on Depressive Symptoms and Glycated Hemoglobin, Blood Pressure, and Serum Cholesterol Among Patients With Depression and Diabetes in India: The INDEPENDENT Randomized Clinical Trial**. *Jama* 2020, **324**(7):651-662.

157. Atif N, Nazir H, Zafar S, Chaudhri R, Atiq M, Mullany LC, Rowther AA, Malik A, Surkan PJ, Rahman A: **Development of a Psychological Intervention to Address Anxiety During Pregnancy in a Low-Income Country**. *Front Psychiatry* 2019, **10**:927.

158. Janssens B, Van Damme W, Raleigh B, Gupta J, Khem S, Soy Ty K, Vun M, Ford N, Zachariah R: **Offering integrated care for HIV/AIDS, diabetes and hypertension within chronic disease clinics in Cambodia**. *Bull World Health Organ* 2007, **85**(11):880-885.

159. Aguiar PM, da Silva CHP, Chiann C, Dórea EL, Lyra DP, Jr., Storpirtis S: **Pharmacist-physician collaborative care model for patients with uncontrolled type 2 diabetes in Brazil: results from a randomized controlled trial**. *J Eval Clin Pract* 2018, **24**(1):22-30.

160. Aydin K, Isildak M, Karakaya J, Gürlek A: **Change in amputation predictors in diabetic foot disease: effect of multidisciplinary approach**. *Endocrine* 2010, **38**(1):87-92.

161. Benzecry SG, Leite HP, Oliveira FC, Santana EMJF, de Carvalho WB, Silva CM: **Interdisciplinary approach improves nutritional status of children with heart diseases**. *Nutrition* 2008, **24**(7-8):669-674.

162. Chung WW, Chua SS, Lai PS, Chan SP: **Effects of a pharmaceutical care model on medication adherence and glycemic control of people with type 2 diabetes**. *Patient Prefer Adherence* 2014, **8**:1185-1194.

163. El-Khoury J, Ghazzaoui R, Ahmad A: **Introducing Specialist Integrated Mental Health Care in Lebanon: The Psychosis Recovery Outreach Program**. *Psychiatr Serv* 2018, **69**(7):738-740.

164. Li S, Blumenthal JA, Shi C, Millican D, Li X, Du X, Patel A, Gao P, Delong E, Maulik PK *et al*: **I-CARE randomized clinical trial integrating depression and acute coronary syndrome care in low-resource hospitals in China: Design and rationale**. *Am Heart J* 2018, **202**:109-115.

165. Lim PC, Lim K, Embee ZC, Hassali MA, Thiagarajan A, Khan TM: **Study investigating the impact of pharmacist involvement on the outcomes of diabetes medication therapy adherence program Malaysia**. *Pak J Pharm Sci* 2016, **29**(2):595-601.

166. Pinotti J, Vieira Alves Andreotti Tojal ML, Toshihiro Nisida AC, Pinotti M: **Comprehensive health care for women in a public hospital in São Paulo, Brazil**. *Reproductive Health Matters* 2001, **9**(18):69-78.

167. Tutino GE, Yang WY, Li X, Li WH, Zhang YY, Guo XH, Luk AO, Yeung RO, Yin JM, Ozaki R *et al*: **A multicentre demonstration project to evaluate the effectiveness and acceptability of the web-based Joint Asia Diabetes Evaluation (JADE) programme with or without nurse support in Chinese patients with Type 2 diabetes**. *Diabet Med* 2017, **34**(3):440-450.

168. Yesil S, Akinci B, Bayraktar F, Havitcioglu H, Karabay O, Yapar N, Demirdover C, Yener S, Yalcin M, Comlekci A *et al*: **Reduction of major amputations after starting a multidisciplinary diabetic foot care team: single centre experience from Turkey**. *Exp Clin Endocrinol Diabetes* 2009, **117**(7):345-349.

169. Zheng Y, Ding X, Guo Y, Chen Q, Wang W, Zheng Y, Wang S, Ding Y, Ding M: **Multidisciplinary management improves anxiety, depression, medication adherence, and quality of life among patients with epilepsy in eastern China: A prospective study**. *Epilepsy Behav* 2019, **100**(Pt A):106400.

170. Jerene D, Hiruy N, Jemal I, Gebrekiros W, Anteneh T, Habte D, Melese M, Suarez P, Sangiwa G: **The yield and feasibility of integrated screening for TB, diabetes and HIV in four public hospitals in Ethiopia**. *Int Health* 2017, **9**(2):100-104.

171. Vodicka EL, Babigumira JB, Mann MR, Kosgei RJ, Lee F, Mugo NR, Okech TC, Sakr SR, Garrison LP, Jr., Chung MH: **Costs of integrating cervical cancer screening at an HIV clinic in Kenya**. *Int J Gynaecol Obstet* 2017, **136**(2):220-228.

172. Were E, Nyaberi Z, Buziba N: **Integrating cervical cancer and genital tract infection screening into mother, child health and family planning clinics in Eldoret, Kenya**. *Afr Health Sci* 2010, **10**(1):58-65.

173. Brown ER, Bartlett J, Chalulu K, Gadama L, Gorman D, Hayward L, Jere Y, Mpinganjira M, Noah P, Raphael M *et al*: **Development of multi-disciplinary breast cancer care in Southern Malawi**. *Eur J Cancer Care (Engl)* 2017, **26**(1).

174. Pfaff C, Singano V, Akello H, Amberbir A, Berman J, Kwekwesa A, Matengeni A, Banda V, Msonko J, Speight C *et al*: **Early experiences in integrating cervical cancer screening and treatment into HIV services in Zomba Central Hospital, Malawi**. *Malawi Med J* 2018, **30**(3):211-214.

175. Adams JL, Almond ML, Ringo EJ, Shangali WH, Sikkema KJ: **Feasibility of nurse-led antidepressant medication management of depression in an HIV clinic in Tanzania**. *Int J Psychiatry Med* 2012, **43**(2):105-117.

176. Palma AM, Rabkin M, Simelane S, Gachuhi AB, McNairy ML, Nuwagaba-Biribonwoha H, Bongomin P, Okello VN, Bitchong RA, El-Sadr WM: **A time-motion study of cardiovascular disease risk factor screening integrated into HIV clinic visits in Swaziland**. *J Int AIDS Soc* 2018, **21**(3):e25099.

177. Rabkin M, Palma A, McNairy ML, Gachuhi AB, Simelane S, Nuwagaba-Biribonwoha H, Bongomin P, Okello VJ, Bitchong RA, El-Sadr WM: **Integrating cardiovascular disease risk factor screening into HIV services in Swaziland: lessons from an implementation science study**. *Aids* 2018, **32 Suppl 1**(Suppl 1):S43-s46.

178. Hopkins KL, Hlongwane KE, Otwombe K, Dietrich J, Cheyip M, Khanyile N, Doherty T, Gray GE: **Level of adult client satisfaction with clinic flow time and services of an integrated non-communicable disease-HIV testing services clinic in Soweto, South Africa: a cross-sectional study**. *BMC Health Serv Res* 2020, **20**(1):404.

179. Jonsson G, Furin J, Jeenah F, Moosa MY, Sivepersad R, Kalafatis F, Schoeman J: **Human rights, mental illness, and HIV: the Luthando Neropsychiatric HIV Clinic in Soweto, South Africa**. *Health Hum Rights* 2011, **13**(2):E64-72.

180. Kajee Z, Harvey J, Zöllner E: **The impact of a diabetes care team on the glycaemic**

**control of paediatric and adolescent patients with**

**type 1 diabetes mellitus at Tygerberg Children’s**

**Hospital**. *South African Journal of Child Health* 2019, **13**(1):12-16.

181. Pillay S, Aldous C: **Introducing a multifaceted approach to the management of diabetes mellitus in resource-limited settings**. *S Afr Med J* 2016, **106**(5):42-43.

182. Ali MK, Singh K, Kondal D, Devarajan R, Patel SA, Shivashankar R, Ajay VS, Unnikrishnan AG, Menon VU, Varthakavi PK *et al*: **Effectiveness of a Multicomponent Quality Improvement Strategy to Improve Achievement of Diabetes Care Goals: A Randomized, Controlled Trial**. *Ann Intern Med* 2016, **165**(6):399-408.

183. Luciano Ede P, Luconi PS, Sesso RC, Melaragno CS, Abreu PF, Reis SF, Furtado RM, Ruivo GF: **Prospective study of 2151 patients with chronic kidney disease under conservative treatment with multidisciplinary care in the Vale do Paraíba, SP**. *J Bras Nefrol* 2012, **34**(3):226-234.

184. Jardim LM, Jardim TV, Souza WK, Pimenta CD, Sousa AL, Jardim PC: **Multiprofessional Treatment of High Blood Pressure in Very Elderly Patients**. *Arq Bras Cardiol* 2017, **108**(1):53-59.

185. Alí A, Giraldo-Cadavid LF, Karpf E, Quintero LA, Aguirre CE, Rincón E, Vejarano AI, Perlaza I, Torres-Duque CA, Casas A: **Frequency of emergency department visits and hospitalizations due to chronic obstructive pulmonary disease exacerbations in patients included in two models of care**. *Biomedica* 2019, **39**(4):748-758.

186. Chan MW, Cheah HM, Mohd Padzil MB: **Multidisciplinary education approach to optimize phosphate control among hemodialysis patients**. *Int J Clin Pharm* 2019, **41**(5):1282-1289.

187. Bukirwa A, Mutyoba JN, Mukasa BN, Karamagi Y, Odiit M, Kawuma E, Wanyenze RK: **Motivations and barriers to cervical cancer screening among HIV infected women in HIV care: a qualitative study**. *BMC Womens Health* 2015, **15**:82.

188. Nakimuli-Mpungu E, Wamala K, Okello J, Alderman S, Odokonyero R, Mojtabai R, Mills EJ, Kanters S, Nachega JB, Musisi S: **Group support psychotherapy for depression treatment in people with HIV/AIDS in northern Uganda: a single-centre randomised controlled trial**. *The Lancet HIV* 2015, **2**(5):e190-e199.

189. Derman W, Schwellnus M, Hope F, Jordaan E, Padayachee T: **Description and implementation of U-Turn Medical, a comprehensive lifestyle intervention programme for chronic disease in the sport and exercise medicine setting: pre-post observations in 210 consecutive patients**. *Br J Sports Med* 2014, **48**(17):1316-1321.
