# Supplementary material for: Understanding integrated service delivery: a scoping review of models for noncommunicable disease and mental health interventions in low-and-middle income countries
Source: BMC Health Serv Res. 2023 Jan 30;23:99. doi: 10.1186/s12913-023-09072-9 (PMC9885613; doi:10.1186/s12913-023-09072-9)
Supplement: Supplementary file 3 — Additional file 3: Supplementary Table 1. Definitions of identified services and disease condition categories (consistent with WHO Integrated health services definition). Supplementary Table 2. Number and percentage of models that include decentralization and/or task shifting stratified by health system level. Supplementary Table 3. Number and percentage of noncommunicable disease or neuropsychiatric models integrated into chronic infection or MCH/PHC models stratified by HIV prevalence in the country. Supplementary Table 4. Number and percentage of common and severe condition categories reported in study models within each country income group. Supplementary Table 5. Number and percentage of studies reporting how care is paid for reported in each study model, stratified by health system level. Supplementary Table 6. Number and percentage of additional domains identified in community-based studies (N=55) including if model was mobile, compensation of primary provider, and primary provider effort. [file 12913_2023_9072_MOESM3_ESM.docx]

**Supplementary table 1: Definitions of identified services and disease condition categories (consistent with** **WHO Integrated health services definition)**

| **Service** | **Definition** |
| --- | --- |
| Health promotion | Education centering on social and environmental interventions designed to benefit and protect individual people’s health and quality of life by addressing and preventing the root causes of ill health, not just focusing on treatment and cure |
| Health education | Education centered around an individual's specific condition or disease, including risk factors, prevention, diagnosis, treatment, self-management |
| Screening | Testing people for disease regardless of clinical presentation. This can include large scale population-based screening or opportunistic screening. |
| Referral system | Passage of patients from one part of the health system to another. This can include referrals up to higher levels of the health system, lateral moves, linkage of stable patients to lower levels of the health system or any combination of the above. |
| Initial diagnosis | Testing and providing a diagnosis for people at risk for a disease |
| Adherence support | Education, intervention, or other support to help patients adhere to their treatment plans |
| Peer group facilitation | Health care professionals enabling peer group support, either for adherence, education or other self-management |
| Home-based care | Care provided for patients at home, often for severe conditions and end of life care |
| Home visits | Visits to patient’s homes for screening, follow-up, monitoring, or medication delivery |
| Acute care | Medical support provided for a new or recurrent condition which has an abrupt onset |
| Psychotherapy | Treating mental health conditions through talking with a trained mental health provider |
| Medication dispensing | Delivering prescribed medicines without dose adjustment |
| Patient follow-up | Repeated interaction with the health system after an initial treatment plan is established. |
| Monitoring | Follow up of patients including vital signs, physical examination, laboratory data, pathology, imaging reports |
| Medication management | Initiation, cessation, or dose adjustment prescribed by a medical provider |
| **Disease condition category** | **Conditions included** |
| Common NCDs | Stage 1-2 hypertension, hyperlipidemia, CVD risk, type 2 diabetes, mild-to-moderate COPD and asthma, mild-to-moderate CKD, chronic respiratory disease, |
| Severe NCDs | Type 1 and insulin-dependent diabetes, RHD, heart failure e.g. due to cardiomyopathies or hypertensive heart disease, acute coronary syndrome, congenital heart disease, sickle cell disease, cancers, liver cirrhosis, end-stage kidney disease, other end-of-life conditions, hypothyroidism, severe (malignant or stage 3) hypertension |
| Common neuropsychiatric | Depression, dementia, epilepsy, anxiety, substance abuse |
| Severe neuropsychiatric | Schizophrenia, psychosis, suicidality, bipolar disorder, PTSD, Alzheimer’s, Parkinson’s |
| Chronic infectious | HIV, TB |
| Acute infection | Malaria, pneumonia, diarrhea, sexually transmitted infections |
| Maternal and child health | Pregnancy and maternal disorders, vaccine-preventable childhood illness, anemia, undernutrition |
| Sense organ | E.g. vision disorders |

**Supplementary table 2: Number and percentage of models that include decentralization and/or task shifting stratified by health system level**

| **Health system level** | **Decentralization**  **n (%)** | **Task shifting**  **n (%)** |
| --- | --- | --- |
| Community (N=55) | 18 (33) | 12 (22) |
| Health center (N=93) | 17 (18) | 34 (37) |
| Secondary level (N=31) | 7 (23) | 12 (39) |
| Tertiary level (N=30) | 0 (0) | 5 (17) |
| Specialty outpatient clinic (N=10) | 1 (10) | 1 (10) |

**Supplementary table 3: Number and percentage of noncommunicable disease or neuropsychiatric models integrated into chronic infection or MCH/PHC models stratified by HIV prevalence in the country**

| **HIV prevalence** | **Chronic infection**  **n (%)** | **MCH and PHC**  **n (%)** |
| --- | --- | --- |
| Low (<1%) (N=91) | 2 (2) | 23 (25) |
| Medium (1-5%) (N=30) | 12 (40) | 16 (53) |
| High (>5%) (N=52) | 35 (67) | 14 (27) |

MCH=maternal and child health, PHC conditions addressed through “Primary health care” not otherwise specified

**Supplementary table 4: Number and percentage of common and severe condition categories reported in study models within each country income group**

|  | **Country income group** | | | |
| --- | --- | --- | --- | --- |
| **Condition category** | **LIC**  **N=29**  **n (%)** | **LMIC**  **N=67**  **n (%)** | **UMIC**  **N=88**  **n (%)** | **Mixed**  **N=4**  **n (%)** |
| Common NCDs | 17 (59) | **41 (62)** | **58 (66)** | 2 (50) |
| Common NPs | **9 (31)** | **21 (31)** | 21 (24) | 2 (50) |
| Severe NCDs | **12 (41)** | 17 (25) | 13 (15) | 0 (0) |
| Severe NPs | **3 (10)** | 5 (7) | 6 (7) | 1 (25) |

NCD=noncommunicable disease, NP=neuropsychiatric

Bolded figure represents income level group with the highest percentage of studies addressing particular condition category

**Supplementary table 5: Number and percentage of studies reporting how care is paid for reported in each study model, stratified by health system level**

| **Financing** | **Community**  **N=55**  **n (%)** | **Health center**  **N=93**  **n (%)** | **Secondary level**  **N= 31**  **n (%)** | **Tertiary level**  **N=30**  **n (%)** | **Specialty outpatient clinic**  **N=10**  **n (%)** |
| --- | --- | --- | --- | --- | --- |
| Free at point of care | 16 (29) | 19 (20) | 16 (52) | 5 (17) | 1 (10) |
| Co-pay | 0 (0) | 3 (3) | 1 (3) | 1 (3) | 0 (0) |
| Out of pocket | 1 (2) | 7 (8) | 0 (0) | 2 (7) | 0 (0) |
| Not specified | 38 (69) | 64 (29) | 14 (45) | 22 (73) | 9 (90) |

**Supplementary table 6: Number and percentage of additional domains identified in community-based studies (N=55) including if model was mobile, compensation of primary provider, and primary provider effort**

| **Mobile** | **n (%)** |
| --- | --- |
| Yes | 10 (18) |
| No | 45 (82) |
| **Compensation of primary provider** | **n (%)** |
| Volunteer | 9 (16) |
| Fee-for-service | 2 (4) |
| Salaried | 6 (11) |
| Unspecified/not applicable | 38 (69) |
| **Effort of primary provider** | **n (%)** |
| Part-time | 9 (16) |
| Full-time | 7 (13) |
| Unspecified/not applicable | 39 (71) |
